# ﻿Review of the *Lycoceruspallidulus* group (Coleoptera, Cantharidae), with descriptions of six new species from China

**DOI:** 10.3897/zookeys.1176.107858

**Published:** 2023-08-29

**Authors:** Younan Wang, Haoyu Liu, Xingke Yang, Yuxia Yang

**Affiliations:** 1 Key Laboratory of Zoological Systematics and Application, School of Life Science, Institute of Life Science and Green Development, Hebei University, Baoding 071002, Hebei Province, China Hebei University Baoding China; 2 Key Laboratory of Zoological Systematics and Evolution, Institute of Zoology, Chinese Academy of Sciences, Beijing, China Key Laboratory of Zoological Systematics and Evolution, Institute of Zoology, Chinese Academy of Sciences Beijing China

**Keywords:** Alpha taxonomy, *Lycocerus*, Oriental region, soldier beetles

## Abstract

The *Lycoceruspallidulus* subgroup, originally placed in the *L.maculicollis* group, is suggested as an independent species group herein and its diagnosis is redefined. Ten previously known species of *Lycocerus* are attributed to this group, including *L.centrochinensis* (Švihla, 2004), *L.genaemaculatus* (Wittmer, 1951), *L.hubeiensis* (Švihla, 2004), *L.kubani* (Švihla, 2004), *L.zdeneki* (Švihla, 2004), *L.bilineatus* (Wittmer, 1995), *L.jelineki* (Švihla, 2004), *L.putzi* Švihla, 2011, *L.pictipennis* (Wittmer, 1995), and *L.curvatus* (Wittmer, 1995). Additionally, six new species of this group are described from China, including *L.laterophysus***sp. nov.**, *L.flavipennis***sp. nov.**, *L.putzimimus***sp. nov.**, *L.maoershanensis***sp. nov.**, *L.chongqingensis***sp. nov.**, and *L.bispermathecus***sp. nov.** These species are illustrated with photographs of habitus, aedeagi, abdominal sternites VIII, and reproductive systems of female. In addition, an identification key and a distribution map of the *L.pallidulus* group are provided.

## ﻿Introduction

*Lycocerus* Gorham, 1889 sensu Okushima (2005) is one of the largest genera in Cantharidae, with more than 300 species worldwide (Liu et al. 2022). No subgenera could be defined in the genus (Okushima 2005), but 16 species-groups have been proposed to date (Okushima 2005; Okushima and Brancucci 2008; Okushima and Yang 2013; Yang et al. 2014; Hsiao and Okushima 2015, 2016; Hsiao et al. 2016; Okushima and Hsiao 2017, 2021; Xi et al. 2021a, b, 2022; Wang et al. 2022). Among these species-groups, *L.maculicollis* group was proposed by Okushima (2005), originally contained three subgroups, including *L.ryukyuanus* subgroup, *L.maculicollis* subgroup and *L.pallidulus* subgroup. This species-group was characterized by median lobe of male genitalia without dorsal process, short spermathecal duct, spermatheca with one spiral tube which is relatively short and stout and gradually expanded to the base (Okushima 2005). However, the monophyly of *L.maculicollis* group was frequently not recovered, with the *L.pallidulus* subgroup in a distant position from *L.maculicollis* subgroup in the morphological phylogenetic trees (Hsiao 2021; Wang et al. 2022; Xi et al. 2022).

Prior to this study, three taxa were included in *L.pallidulus* subgroup, including *L.pallidulus* (Wittmer, 1995), *L.guerryi* (Pic, 1906), and *L.guerryiatroapicipennis* (Pic, 1914) (Okushima 2005). In our taxonomic study on the Chinese *Lycocerus*, we found some previously known species should be placed in this group, meanwhile, we discovered some hitherto unknown species. With more species added, we discovered that some species did not agree perfectly with the conventional characteristics, also referred to in the previous phylogenetic results and these need further work.

## ﻿Materials and methods

The studied material is preserved in the following collections:

**CWNU** China West Normal University, Nanchong, China;

**IZAS**Institute of Zoology, Chinese Academy of Sciences, Beijing, China;

**MHBU** Museum of Hebei University, Baoding, China;

**MNHN**Muséum national d’Histoire naturelle, Paris, France;

**NHMB**Naturhistorisches Museum Basel, Switzerland;

**NMPC**National Museum, Prague, Czech Republic;

**NWAFU** Northwest Agriculture & Forestry University, Yangling, China;

**ZFMK** Zoological Research Museum Alexander Koenig, Bonn, Germany.

We identified specimens based on examination of the type material and reference to the relevant publications (Wittmer 1995; Švihla 2004, 2011; Okushima 2005), from which we also derived our taxon concepts and taxonomic classification. Morphological terminology used in this study mainly follows that of Okushima (2005) and Kazantsev (2023).

Genitalia of both sexes and abdominal sternites VIII of females were dissected and cleared in 10% NaOH solution, and female genitalia was dyed with hematoxylin. Habitus photos were taken by a Leica M205A stereomicroscope. Line drawings were made using a camera lucida attached to a Nikon SMZ1500 stereomicroscope, then edited in CorelDraw 12 and Adobe Photoshop CS6.13.0.

The label of the specimens in Chinese are transliterated, and the complete label data are cited for the type specimens. The distribution information was collected from the publications (Wittmer 1951, 1995; Švihla 2004, 2011) and the examined material of the present study. The distribution map was prepared by ArcMap 10.8 and edited in Adobe Photoshop CS6.13.0.

## ﻿Results

### ﻿*Lycoceruspallidulus* group

**Figure 1.**
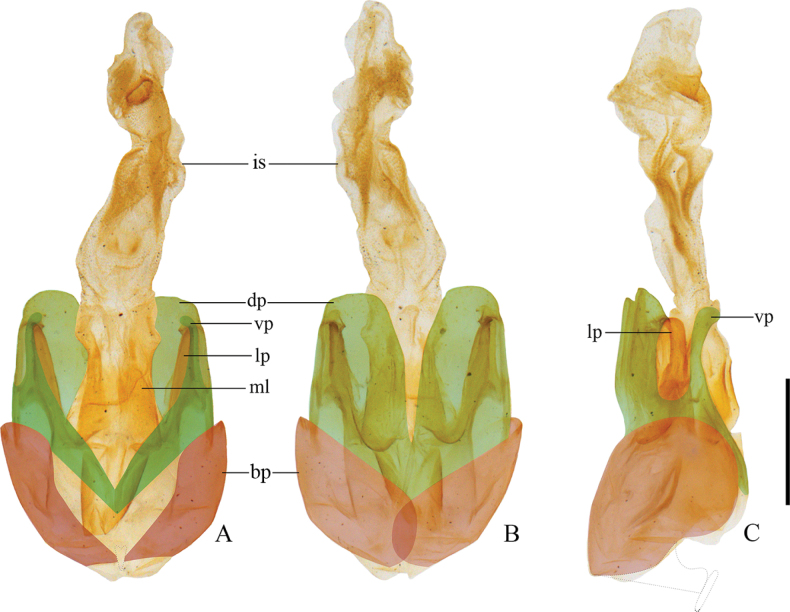
Aedeagus of *Lycoceruscurvatus* (Wittmer, 1995): **A** ventral view **B** dorsal view **C** lateral view. Abbreviations: bp – basal piece; dp – dorsal plate of each paramere; lp – laterophyse; ml – median lobe; vp – ventral process of each paramere; is – inner sac. Scale bar: 1.0 mm. The red hues show basal pieces (with middle nodule in ventral part missing), green for parameres, and yellow represents median lobe exhibiting with inner sac and laterophyses.

**Common characteristics.** Body middle-sized (8.0–11.5 mm), slender. Antennae filiform, present or absent with impressions on middle antennomeres in male. Pronotum subquadrate, longer than or nearly as long as wide. Elytra pale yellow or even transparent, sometimes with black longitudinal bands or markings on disc, subparallel-sided. Tarsal claws diverse: if all simple in male, fore and mid-anterior and or posterior claws each with a digitiform tooth at base in female; when fore and mid-anterior claws each with a tooth in male, same in female or both anterior and posterior claws each with a tooth; otherwise, fore and mid-anterior and posterior claws each with a tooth in both sexes; hind claws always simple. Aedeagus (Fig. 1): dorsal plates of parameres separate, each with a keel near lateral margin and located on inner surface, laterophyses well-developed and nearly as long as ventral processes, with apices opposite to the keels of dorsal plates, inner sac of median lobe lengthened apically and nearly as long as tegmen, without dorsal process. Female internal genitalia: vagina stout and abruptly thinned at ventroapical portion into a stout tube, where diverticulum and spermathecal duct arising separately; diverticulum moderately long, thin, and spiral; spermathecal duct short and stout; spermatheca with a spiral tube, often abruptly thinned apically near base, basal portion of spermatheca extended into a short tube, where accessory gland opening, accessory gland longer than spermatheca.

**Included species.***L.pallidulus* (Wittmer, 1995), *L.guerryi* (Pic, 1906), *L.guerryiatroapicipennis* (Pic, 1914), *L.centrochinensis* (Švihla, 2004), *L.genaemaculatus* (Wittmer, 1951), *L.hubeiensis* (Švihla, 2004), *L.jelineki* (Švihla, 2004), *L.putzi* Švihla, 2011, *L.bilineatus* (Wittmer, 1995), *L.zdeneki* (Švihla, 2004), *L.kubani* (Švihla, 2004), *L.curvatus* (Wittmer, 1995), *L.pictipennis* (Wittmer, 1995), *L.laterophysus* sp. nov., *L.flavipennis* sp. nov., *L.putzimimus* sp. nov., *L.maoershanensis* sp. nov., *L.chongqingensis* sp. nov. and *L.bispermathecus* sp. nov.

**Figure 2.**
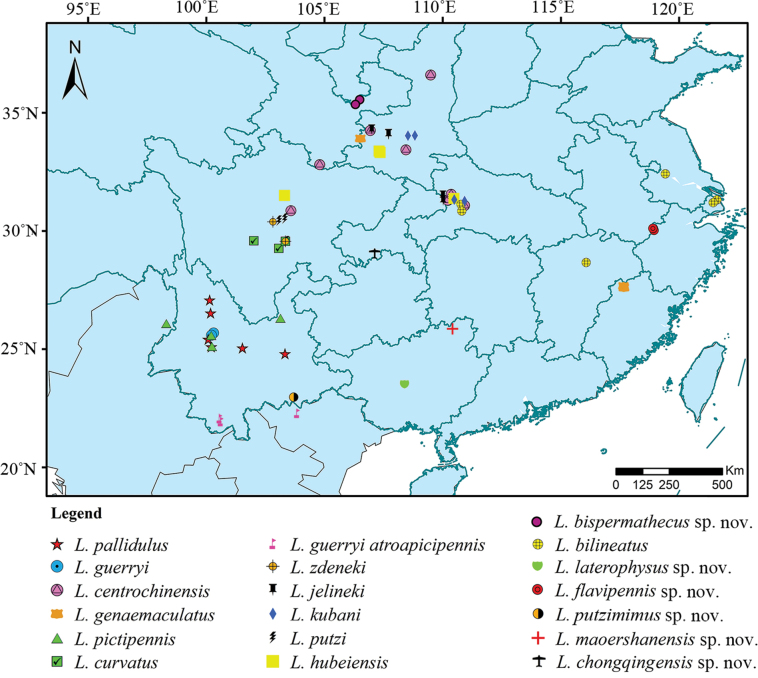
Distribution map of *Lycoceruspallidulus* group.

**Distribution (Fig. 2).** China (Yunnan, Sichuan, Fujian, Jiangsu, Shanghai, Jiangxi, Hubei, Shaanxi, Ningxia, Guangxi, Zhejiang); Vietnam; Myanmar.

#### ﻿ Lycocerus pallidulus

Taxon classificationAnimaliaColeopteraCantharidae

(Wittmer, 1995)

7B2F7FCF-E103-5FF5-9354-E7CB42640831

Athemus (Isathemus) pallidulus Wittmer, 1995: 261, figs 119, 189.
Lycocerus pallidulus : Okushima 2005: 48, figs 11b, 12c, 14b, 22, 74.

##### Type material examined.

***Holotype***: 1♂ (NHMB), Yunnan, Lijiang, 1800 m, 26°53'N, 100°18'E, 23.VI–21.VII.1992, lgt. S. Becvar.

##### Distribution.

China (Yunnan).

##### Remarks.

This species was omitted from the Palaearctic Catalogue by Kazantsev and Brancucci (2007). Both the aedeagus and female internal genitalia of this species have been well illustrated by Okushima (2005).

#### ﻿ Lycocerus guerryi

Taxon classificationAnimaliaColeopteraCantharidae

(Pic, 1906)

10EA4010-FBE2-5684-BBAA-95F1A8C5E378

**Figure 3.**
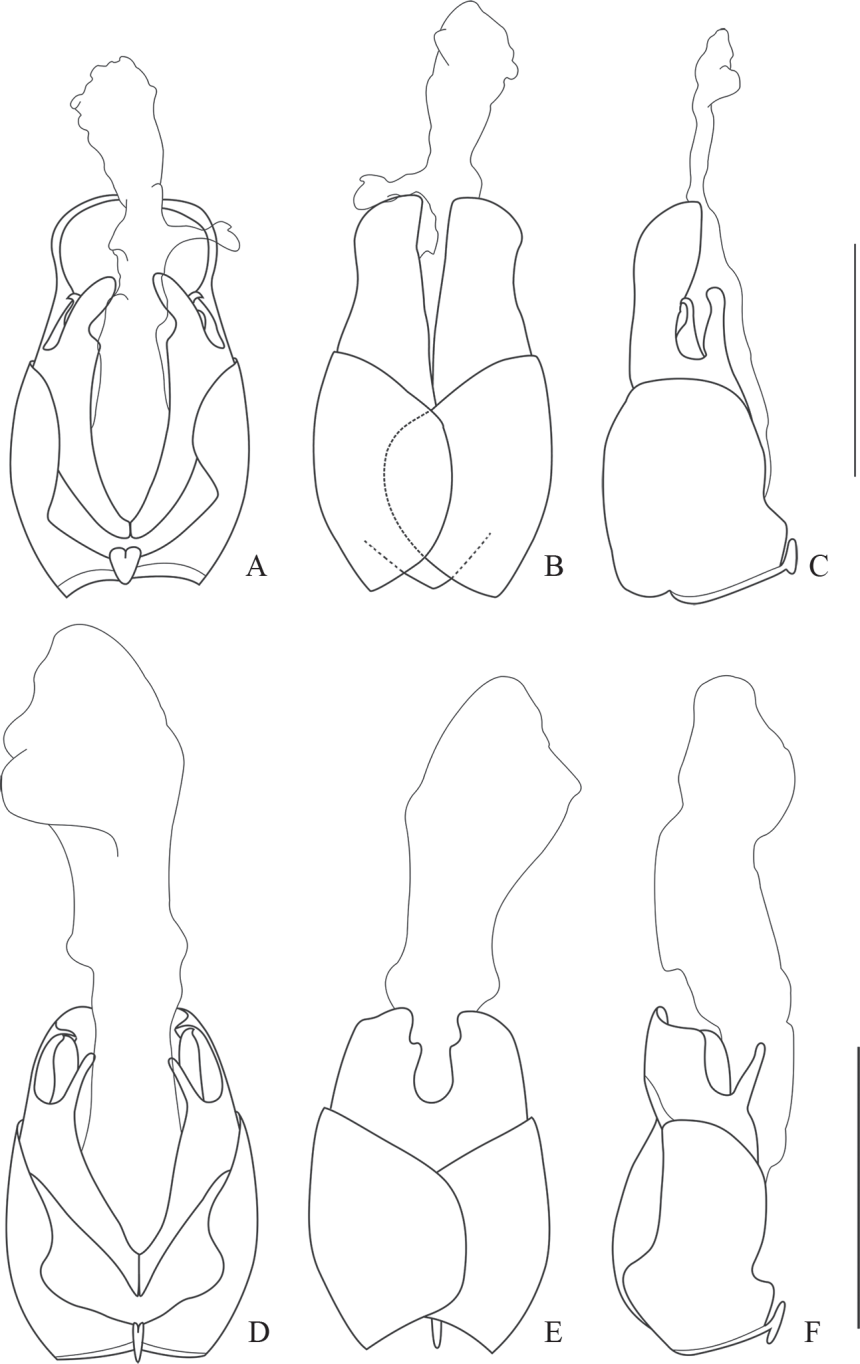
Aedeagus **A–C***Lycocerusguerryi* (Pic, 1906) **D–F***L.centrochinensis* (Švihla, 2004) **A, D** ventral view **B, E** dorsal view **C, F** lateral view. Scale bars: 1.0 mm.

Figs 3A–C , 12C , 16C

Cantharis guerryi Pic, 1906: 83.
Athemus (Isathemus) guerryi : Wittmer 1995: 261.
Lycocerus guerryi : Okushima 2005: 48; Kazantsev and Brancucci 2007: 250.

##### Type material examined.

***Holotype***: 1♂ (MNHN), China, P. Guerry.

##### Non-type material examined.

China: 4♂1♀ (IZAS), Yunnan, Dali, 2100 m, 31.V.1955, leg. L. Wu; 1♀ (IZAS), Yunnan, Dali, 2100 m, 30.V.1955, leg. Bussik; 1♀ (IZAS), Yunnan, Xiaguan, 2050 m, 30.V.1955, leg. S. C. Ha.

##### Descriptive notes.

**Male.** Aedeagus: basal piece nearly as long as dorsal plate of each paramere (Fig. 3A–C); ventral process of each paramere stout and abruptly narrowed near apex, obviously bent inwards in ventral view (Fig. 3A), slightly bent dorsally in lateral view (Fig. 3C); dorsal plates of parameres obviously longer than ventral processes, with lateral margins slightly sinuate in middle, apical margins slightly arcuate and descending outwards in lateral view (Fig. 3B); laterophyse feebly shorter than ventral process, with apex acute and directing dorso-outwards (Fig. 3A); inner sac of median lobe with a stout tube extruding near base (Fig. 3A, B).

**Female.** Internal organ of reproductive system (Fig. 12C): spermatheca nearly as long as diverticulum; accessory gland ~ 2.5× longer than spermatheca.

Abdominal sternite VIII (Fig. 16C): triangular emarginations on both sides and in middle of posterior margin, lateral emarginations slightly deeper and much wider than the middle one, the portions between lateral and middle emarginations wide and obtuse-triangular at apices, slightly extending over apices of latero-apical angles, which are nearly truncated.

##### Distribution.

China (Yunnan).

##### Remarks.

*Cantharisjeanvoinei* Pic, 1927 was listed as a synonym of *L.guerryi* by Kazantsev and Brancucci (2007), which is an obvious error. In fact, the former was synonymized with *L.guerryiatroapicipennis* (Pic, 1914) by Wittmer (1995), but was missing in the Palaearctic Catalogue (Kazantsev and Brancucci 2007).

#### ﻿ Lycocerus guerryi atroapicipennis

Taxon classificationAnimaliaColeopteraCantharidae

(Pic, 1914)

0B02366F-7EC4-598C-BC45-83E54C883315

Cantharis atroapicipennis Pic, 1914:8.
Athemus (Isathemus) guerryi atroapicipennis : Wittmer 1995: 274, fig. 139.
Cantharis jeanvoinei Pic, 1927: 2. Synonymized by Wittmer 1995: 274.
Lycocerus guerryi atroapicipennis : Okushima 2005: 48.

##### Type material examined.

***Holotype*** of *Cantharisatroapicipennis*: 1♂ (MNHN), Lao Kay.

***Holotype*** of *Cantharisjeanvoinei*: 1♀ (MNHN), Tonkin, Chapa, 7.V.1918, Eanvoine.

##### Non-type material examined.

China: 1♂ (IZAS), Yunnan, Menlongbanna, Mengsong, 1600 m, 22.IV.1958, leg. C. B. Hong; 1♂ (IZAS), same data as the preceding, 23.IV.1958; 1♂(IZAS), Yunnan, Menghai, Nannuoshan, 1100 m, 28.IV.1957, leg. F. J. Pu.

##### Distribution.

China (new record: Yunnan); Vietnam.

##### Remarks.

The aedeagus of this subspecies is very similar to that of *L.guerryiguerryi*, but its elytra are darkened at apices enabling external identification. The body coloration is constant, and the elytra of the individuals from northern Yunnan are uniformly pale yellow (*L.guerryiguerryi*), while those from southern Yunnan and adjacent area (northern Vietnam) are always darkened at apices (*L.guerryiatroapicipennis*), so they are kept as two subspecies at the moment. Here, *L.guerryiatroapicipennis* is recorded in the Chinese fauna for the first time.

#### ﻿ Lycocerus centrochinensis

Taxon classificationAnimaliaColeopteraCantharidae

(Švihla, 2004)

47072CC6-CB03-529B-A558-6E5F37ED5EEC

Figs 3D–F , 12B , 16B

Athemus (s.str.) centrochinensis Švihla, 2004: 182, figs 83–85, 202.
Lycocerus centrochinensis : Kazantsev and Brancucci 2007: 250.

##### Type material examined.

***Holotype***: 1♂ (NMPC), China, Shaanxi, Quing Ling Shan mts, m rd. Baoji-Taibai, 35 km S Baoji, 21–23.VI.1998, lgt. O. Šafránek & M. Trýzna.

##### Non-type material examined.

China: 2♂1♀ (MHBU), Sichuan, Wolong, 6–7.VIII.2004, leg. X. J. Yang & H. R. Hua; 1♂1♀ (MHBU), Gansu, Wenxian, Huangtuling, 2350 m, 8.VII.2003, leg. Y. B. Ba & Y. Yu; 1♂ (MHBU), Hubei, Dalaoling Nature Reserve, 1200 m, 9.VII.2011, leg. X. L. Liang; 1♀ (MHBU), same locality as the preceding, 11.VII.2011, leg. X. Liao; 1♂ (MHBU), Hubei, Shennongjia, Muyuzhen, 1200 m, 12.VII.2004, leg. S. Q. Xu; 1♀ (MHBU), same data as the preceding, leg. F. L. Zou; 1♀ (MHBU), Hubei, Shennongjia, Wenshui Forestry, 1700–2000 m, 16.VII.2003, leg. C. Gui; 1♂2♀ (MHBU), Shaanxi, Fengxian, Jialing, Jiangyuan, 13.VII.2012, leg. G. D. Ren; 1♂4♀ (MHBU), Shaanxi, Ningshaan, Huoditang, 1505 m, 33.434126°N, 108.448091°E (DD), 15.VIII.2013, leg. X. C. Zhu & Y. Tian.

##### Descriptive notes.

**Male.** Aedeagus: basal piece slightly longer than dorsal plate of each paramere (Fig. 3D–F); ventral process of each paramere thin and straight, approaching to each other in ventral view (Fig. 3D) and inclining ventrally in lateral view (Fig. 3F); dorsal plates of parameres slightly longer than ventral processes (Fig. 3D, F), with inner margins abruptly diverging near middle, apical margins slightly arcuate and descending outwards in dorsal view (Fig. 3E); laterophyse feebly longer than ventral process, with apices acute and directing dorso-outwards (Fig. 3D, F).

**Female.** Internal organ of reproductive system (Fig. 12B): spermatheca nearly as long as diverticulum; accessory gland ~ 3.0× longer than spermatheca.

Abdominal sternite VIII (Fig. 16B): rounded emargination in middle and triangular emarginations on both sides of posterior margin, lateral emarginations obviously deeper than and nearly as wide as the middle one, the portions between lateral and middle emarginations moderately wide and right-angled at apices, obviously extending over apices of latero-apical angles, which are narrowly triangular.

##### Distribution.

China (Shaanxi, Hubei, Sichuan, Gansu).

##### Remarks.

Li et al. (2015) provided an illustration of female internal genitalia for *L.centrochinensis*, which is of an unknown species. Additionally, the distribution range of this species is expanded, with geographic records added from Sichuan and Gansu provinces.

#### ﻿ Lycocerus genaemaculatus

Taxon classificationAnimaliaColeopteraCantharidae

(Wittmer, 1951)

00B1F632-974B-56AF-91B8-23FF6E23F1F8

**Figure 4.**
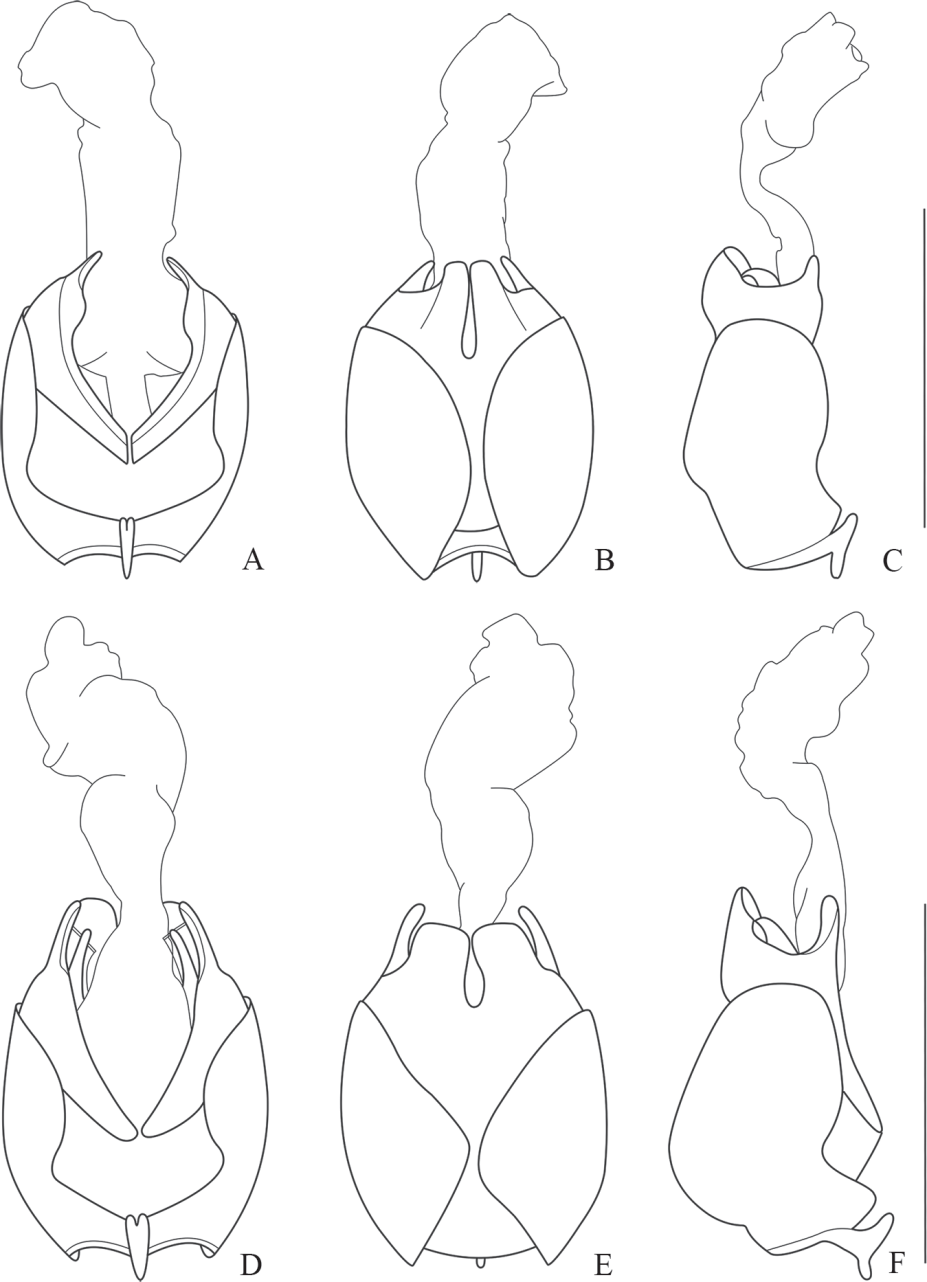
Aedeagus **A–C***Lycocerusgenaemaculatus* (Wittmer, 1951) **D–F***L.hubeiensis* (Švihla, 2004) **A, D** ventral view **B, E** dorsal view **C, F** lateral view. Scale bars: 1.0 mm.

Figs 4A–C , 12D , 16D

Athemus genaemaculatus Wittmer, 1951: 100.
Athemus (Isathemus) genaemaculatus : Wittmer 1995: 259.
Lycocerus genaemaculatus : Kazantsev and Brancucci 2007: 250.

##### Type material examined.

***Holotype***: 1♂ (ZFMK), Kuatun (Fukien), 2300 m, 27.40n, Br. 117.40ö, 20.V.1938, L. J. Klapperich. ***Paratypes***: 1♀ (NHMB), Fukien (Fujian), Kuatun, 21.IV.1946, lgt. Tschung Sen; 1♀ (NHMB), Fukien (Fujian), Kuatun, 2300 m, 27°40'N, 117°40'E (DDM), 13.V.1938, lgt. J. Klapperich.

##### Non-type material examined.

China: 1♂ (IZAS), Fujian, Jianyang, Huangkeng, Aotou, 950 m, 2.V.1960, leg. Y. R. Zhang; 1♀ (IZAS), Fujian, Jianyang, Huangkeng, Guilin, 270–340 m, 8.IV.1960, leg. Y. R. Zhang; 1♀ (IZAS), same locality as the preceding, 290–320 m, 12.IV.1960, leg. F. J. Pu.

##### Descriptive notes.

**Male.** Aedeagus: basal piece distinctly longer than dorsal plate of each paramere (Fig. 4A–C); ventral process of each paramere short and thin, nearly straight and approaching to each other in ventral view (Fig. 4A), slightly bent dorsally in lateral view (Fig. 4C); dorsal plates of parameres as long as ventral process (Fig. 4B, C), with outer margins abruptly converging apically, apical margins subrounded in dorsal view (Fig. 4B); laterophyse feebly shorter than ventral process, with apices acute and markedly directing dorso-outwards (Fig. 4C).

**Female.** Internal organ of reproductive system (Fig. 12D): spermatheca nearly as long as diverticulum; accessory gland ~ 3.0× longer than spermatheca.

Abdominal sternite VIII (Fig. 16D): triangular emargination in middle and subrounded emarginations on both sides of posterior margin, lateral emarginations slightly deeper and obviously wider than the middle one, the portions between lateral and middle emarginations narrow and acute at apices, obviously extending over apices of latero-apical angles, which are widely triangular.

##### Distribution.

China (Fujian).

##### Remarks.

The aedeagus, abdominal sternite VIII, and internal genitalia of female are illustrated for the first time for this species herein.

#### ﻿ Lycocerus hubeiensis

Taxon classificationAnimaliaColeopteraCantharidae

(Švihla, 2004)

AEFAF9C6-1BA1-5634-9C07-AF06801AAF55

Figs 4D–F , 12A , 16A

Athemus (Isathemus) hubeiensis Švihla, 2004: 191, figs 124, 125.
Lycocerus hubeiensis : Kazantsev and Brancucci 2007: 250.

##### Type material examined.

***Holotype***: 1♂ (NMPC), China, Hubei, Dashennongjia mts., 2000–3000 m, 31°05'N, 103°03'E (DDM), 21–24.VI.2001, lgt. O. Šafánek.

##### Non-type material examined.

China: 1♂ (MHBU), Shaanxi, Yangxian, Huayanghanba, 1014 m, 33°32'52"N, 107°35'5"E (DMS), 6.V.2017, leg. H. Y. L iu; 1♀ (MHBU), Shaanxi, Yangxian, Huayangzhen, Yantou, 1206 m, 33°38'29"N, 107°31'6.9"E (DMS), 7.VIII.2017, leg. H. Y. Liu & X. D. Zhang; 1♀ (MHBU), Hubei, Chaoshuihe, 23.V. 2019, leg. P. Wang.

##### Descriptive notes.

**Male.** Aedeagus: basal piece distinctly longer than dorsal plate of each paramere (Fig. 4D–F); ventral process of each paramere nearly straight, feebly bent inwards in ventral view (Fig. 4D) and vertical in lateral view (Fig. 4F); dorsal plates of parameres feebly longer than ventral processes (Fig. 4D, F), with outer margin obviously abruptly converging apically, apical margins truncate in dorsal view (Fig. 4E); laterophyse slightly shorter than ventral process, with apices acute and appreciably directing dorso-inwards (Fig. 4F).

**Female.** Internal organ of reproductive system (Fig. 12A): spermatheca nearly as long as diverticulum; accessory gland ~ 2.5× longer than spermatheca.

Abdominal sternite VIII (Fig. 16A): triangular emargination in middle and narrowly rounded emarginations on both sides of posterior margin, lateral emarginations obviously deeper and feebly wider than the middle one, the portions between lateral and middle emarginations moderately wide and right-angled at apices, obviously extending over apices of latero-apical angles, which are narrowly rounded.

##### Distribution.

China (Hubei, Shaanxi).

##### Remarks.

The aedeagus of this species was illustrated only in ventral and lateral views by Švihla (2004). Here, the aedeagus is illustrated in ventral, dorsal, and lateral views, and the abdominal sternite VIII and internal genitalia of the female are illustrated for the first time. Additionally, the distribution range of this species is expanded, with geographic records added from Shaanxi Province.

#### ﻿ Lycocerus kubani

Taxon classificationAnimaliaColeopteraCantharidae

(Švihla, 2004)

ECC428E9-8064-5445-A4AB-BE1F1034E223

**Figure 5.**
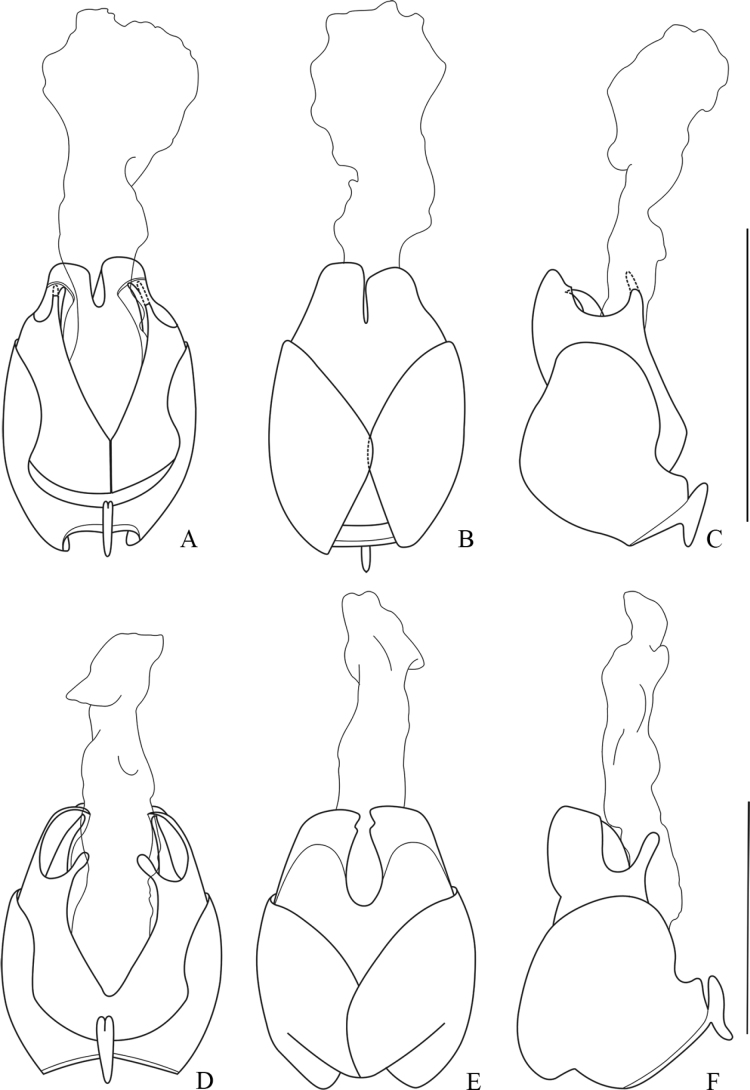
Aedeagus **A–C***Lycoceruskubani* (Švihla, 2004) **D–F***L.zdeneki* (Švihla, 2004) **A, D** ventral view **B, E** dorsal view **C, F** lateral view. Scale bars: 1.0 mm.

Figs 5A–C , 14C , 16G

Athemus (Isathemus) kubani Švihla, 2004: 190, figs 122, 123, 207.
Lycocerus kubani : Kazantsev and Brancucci 2007: 251.

##### Type material examined.

***Holotype***: 1♂ (NMPC), China, Shaanxi, Haozhenzi env., 1350–2000m, 14–24.VI.1999, lgt. S. Murzin.

##### Non-type material examined.

China: 1♂, 1♀ (IZAS), Hubei, Xingshan, Xiaohekou, 700 m, 11.V.1994, leg. X. K. Yang; 1♀ (IZAS), Hubei, Xingshan, Longmenhe, 1400 m, 16.VI.1993, leg. Z. R. Huang; 1♀ (IZAS), same locality as the preceding, 1310 m, 15.VI.1993, leg. J. Yao.

##### Descriptive notes.

**Male.** Aedeagus: basal piece obviously longer than dorsal plate of each paramere (Fig. 5A–C); ventral process of each paramere thin and nearly straight, approaching to each other in ventral view (Fig. 5A) and inclining dorsally in lateral view (Fig. 5C); dorsal plates of parameres feebly longer than ventral processes (Fig. 5A, C), with outer margins converging apically in dorsal view, apical margins truncate at apices (Fig. 5B); laterophyse feebly shorter than ventral process, with apices acute and directing dorso-outwards (Fig. 5C).

**Female.** Internal organ of reproductive system (Fig. 14C): spermatheca feebly longer than diverticulum; accessory gland ~ 1.2× longer than spermatheca.

Abdominal sternite VIII (Fig. 16G): triangular emargination in middle and rounded emarginations on both sides of posterior margin, lateral emarginations obviously deeper and wider than the middle one, the portion between lateral and middle emarginations narrow and acute at apices, obviously extending over apices of latero-apical angles, which are rounded.

##### Distribution.

China (Shaanxi, Hubei).

##### Remarks.

The aedeagus of this species was illustrated only in ventral and lateral views by Švihla (2004). Here, the aedeagus is illustrated in ventral, dorsal, and lateral views, and female internal genitalia is illustrated for the first time. Additionally, the distribution range of this species is expanded, with geographic records added from Hubei province.

#### ﻿ Lycocerus zdeneki

Taxon classificationAnimaliaColeopteraCantharidae

(Švihla, 2004)

77B7CB3E-398A-595B-AA64-B38418C64477

Figs 5D–F , 14B , 16F

Athemus (Isathemus) zdeneki Švihla, 2004: 192, figs 126–128.
Lycocerus zdeneki : Kazantsev and Brancucci 2007: 254.

##### Type material examined.

***Holotype***: 1♂ (NMPC), China, Sichuan, Baoxing, 100 km N of Yaan, 12–14.VII.1995, lgt. Zd. Jindra.

##### Non-type material examined.

China: 1♂ (IZAS), Sichuan, Emeishan, Xixiangdi, 1800–2000 m, 25.VI.1957, leg. F. X. Zhu; 1♀ (IZAS), same locality as the preceding, 12.VII.1957, leg. F. X. Zhu.

##### Descriptive notes.

**Male.** Aedeagus: basal piece slightly longer than dorsal plate of each paramere (Fig. 5D–F); ventral process of each paramere slender and slightly expanded at apices, nearly straightly and approaching to each other in ventral view (Fig. 5D), inclining ventrally in lateral view (Fig. 5F); dorsal plates of parameres feebly longer than ventral process (Fig. 5D, F), with inner margins feebly emarginate at inner apical angles, outer margins slightly converging apically in dorsal view (Fig. 5E); laterophyse slightly longer than ventral process, with apices acute and appreciably directing dorso-outwards (Fig. 5F).

**Female.** Internal organ of reproductive system (Fig. 14B): spermatheca slightly longer than diverticulum; accessory gland slightly longer than spermatheca.

Abdominal sternite VIII (Fig. 16F): right-triangular emargination in middle and subrounded emarginations on both sides of posterior margin, lateral emarginations feebly deeper and wider than the middle one, the portions between lateral and middle emarginations wide and wide-triangular at apices, feebly extending over apices of latero-apical angles, which are truncated.

##### Distribution.

China (Sichuan).

##### Remarks.

Only the apical parts of the aedeagus of this species was illustrated by Švihla (2004). In this work, the aedeagus is illustrated in general views, and the abdominal sternite VIII and internal genitalia of the female are illustrated for the first time.

#### ﻿ Lycocerus bilineatus

Taxon classificationAnimaliaColeopteraCantharidae

(Wittmer, 1995)

AA769D44-A64B-50B7-95CC-35327A576985

**Figure 6.**
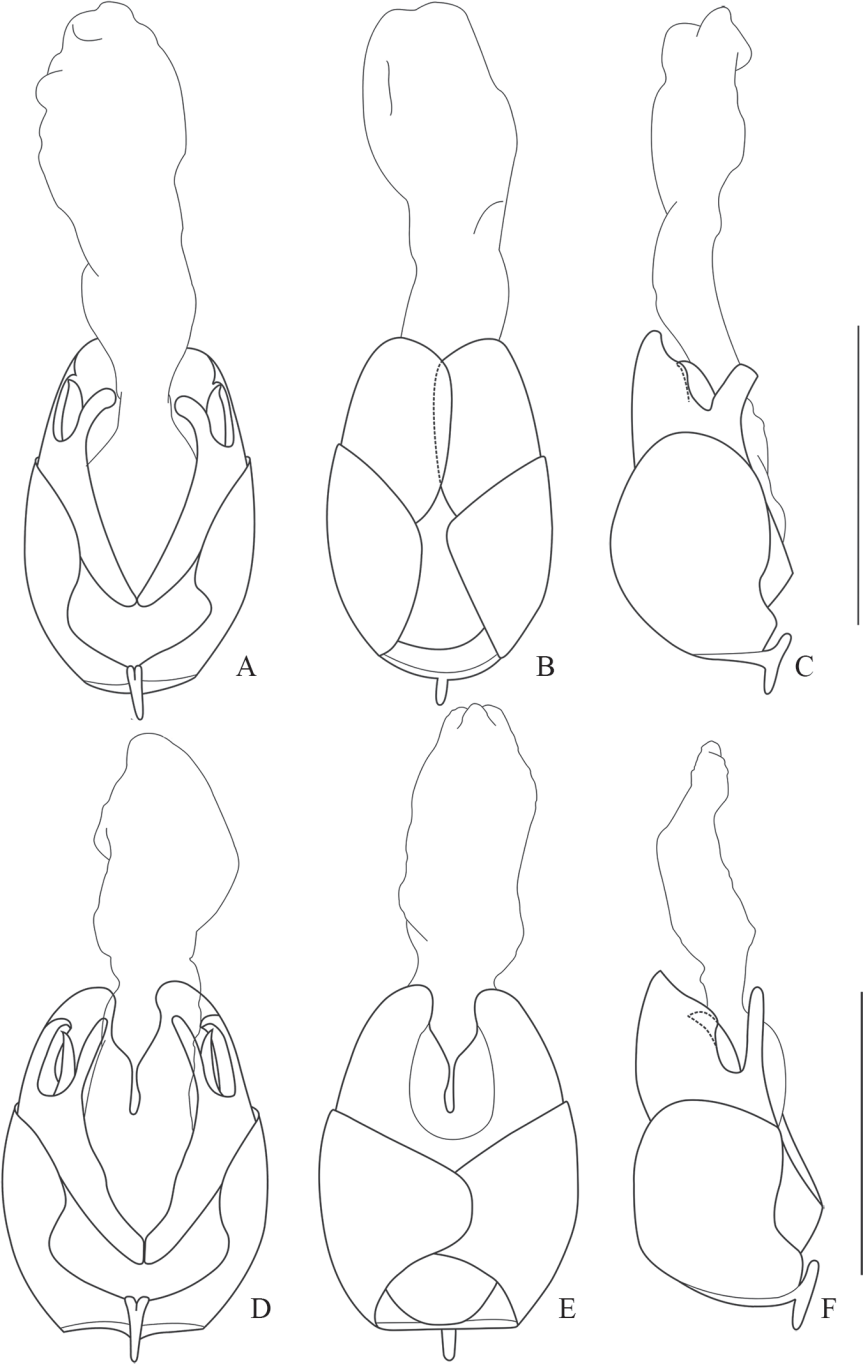
Aedeagus **A–C***Lycocerusbilineatus* (Wittmer, 1995) **D–F***L.jelineki* (Švihla, 2004) **A, D** ventral view **B, E** dorsal view **C, F** lateral view. Scale bars: 1.0 mm.

Figs 6A–C , 13A , 17A

Athemus (Isathemus) bilineatus Wittmer, 1995: 275, figs 140, 141.
Athemus (s.str.) amplus Wittmer, 1995: 278, figs 146, 147, 203.
Lycocerus amplus : Kazantsev and Brancucci 2007: 249. Synonymized by Yang et al. 2013: 10, fig. 8.
Lycocerus bilineatus : Kazantsev and Brancucci 2007: 249; Yang et al. 2013: 10, fig. 7.

##### Type material examined.

See Yang et al. (2013).

##### Non-type material examined.

China: 2♂ , 1♀ (IZAS), Jiangxi, date and collector unknown; 1♀ (IZAS), Shanghai, 1947, leg. Marist Brothers.

##### Descriptive notes.

**Male.** Aedeagus: basal piece feebly longer than dorsal plate of each paramere (Fig. 6A–C); ventral process of each paramere stout and obviously bent inwards apically in ventral view (Fig. 6A), inclining ventrally in lateral view (Fig. 5C); dorsal plates of parameres obviously longer than ventral processes (Fig. 5A, C), with apical margins rounded (Fig. 5B); laterophyse feebly longer than ventral process, with apices acute and directing dorso-outwards (Fig. 5A, C).

**Female.** Internal organ of reproductive system (Fig. 13A): spermatheca feebly longer than diverticulum; accessory gland nearly as long as spermatheca.

Abdominal sternite VIII (Fig. 17A): triangular emarginations in middle and on both sides of posterior margin, lateral emarginations obviously deeper and wider than the middle one, the portion between lateral and middle emarginations wide and right-angled at apices, obviously extending over apices of latero-apical angles, which are narrowly rounded.

##### Distribution.

China (Jiangsu, Shanghai, Jiangxi, Hubei).

##### Remarks.

The aedeagus of this species was illustrated only in ventral and lateral views by Wittmer (1995). Herein, the aedeagus is illustrated in ventral, dorsal, and lateral views, and the female internal genitalia is illustrated for the first time.

#### ﻿ Lycocerus jelineki

Taxon classificationAnimaliaColeopteraCantharidae

(Švihla, 2004)

CD7F4595-524E-5CFF-9D0E-78216AC6F6FD

Figs 6D–F , 13B , 17B

Athemus (Andrathemus) jelineki Švihla, 2004: 189, figs 109–111, 204.
Lycocerus jelineki : Kazantsev and Brancucci 2007: 251.

##### Type material examined.

***Holotype***: 1♂ (NMPC), China, Shaanxi, Qinling mts. m rd. Baoji-Taibai, 35 km S Baoji, 21–23.VI.1998, lgt. O. Šafránek & M. Trýzna.

##### Non-type material examined.

China: 1♂, W Hubei prov. Dashennongjia Nat. Res. Muyu, E slope, 2000 m, 12–15.VI.1997, leg. Bolm; 1♂ (MHBU), Hubei, Shennongjia, Pingqian, 1576 m, 31°28'08.0"N, 110°02'23.4"E (DMS), 4–7.VII.2014, leg. Y.B. Ba & S. Y. Tang; 1♂ (NWAFU), Shaanxi, Taibaishan, Haopingsi, 1200 m, 31.V.1981, collector unknown; 1♂ (NWAFU), Shaanxi, Taibaishan, Zhongshansi, 1500 m, 9.VI.1981, collector unknown; 1♀ (NWAFU), Shaanxi, Taibaishan, Haopingsi, 25.VI.1982, collector unknown; 1♀ (NWAFU), Shaanxi, Taibaishan, Zhongshansi, 400 m, 11.VI.1981, collector unknown.

##### Descriptive notes.

**Male.** Aedeagus: basal piece feebly longer than dorsal plate of each paramere (Fig. 6D–F); ventral process of each paramere nearly slender and approaching to each other in ventral view (Fig. 6D), nearly vertical in lateral view (Fig. 6F); dorsal plates feebly longer than ventral process (Fig. 6D, F), with inner margins abruptly diverging near middle, outer margins slightly converging apically, apical margins subrounded (Fig. 6E); laterophyse slightly shorter than ventral process, with apices acute and appreciably directing dorso-outwards (Fig. 6D, F).

**Female**. Internal organ of reproductive system (Fig. 13B): spermatheca nearly as long as diverticulum; accessory gland ~ 2× longer than spermatheca.

Abdominal sternite VIII (Fig. 17B): rounded emargination in middle and subtriangular emargination on both sides of posterior margin, lateral emarginations deeper and feebly narrower than the middle one, the portion between lateral and middle emarginations wide and widely triangular at apices, extending over apices of latero-apical angles, which are subrounded.

##### Distribution.

China (Shaanxi, Hubei).

##### Remarks.

Sometimes the pronotum and vertex have dark brown spots, in both sexes. Only the apical parts of the aedeagus were illustrated by Švihla (2004). Here, the aedeagus is illustrated in general views, and the female internal genitalia is illustrated for the first time.

#### ﻿ Lycocerus putzi

Taxon classificationAnimaliaColeopteraCantharidae

Švihla, 2011

3BA931E7-CC4C-51CD-92F3-78858DEAB67A

**Figure 7.**
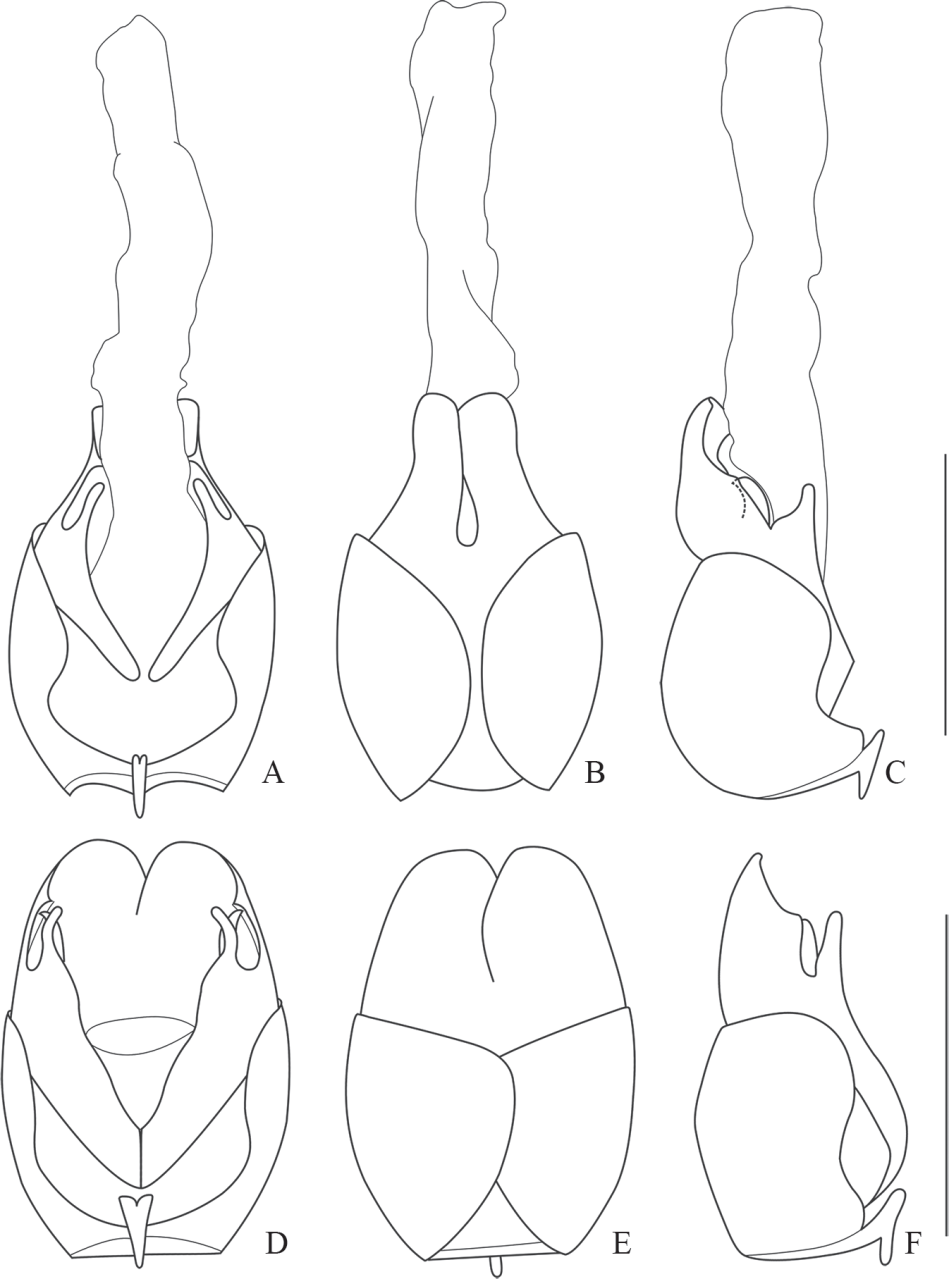
Aedeagus **A–C***Lycocerusputzi* Švihla, 2011 **D–F***L.pictipennis* (Wittmer, 1995) **A, D** ventral view **B, E** dorsal view **C, F** lateral view. Scale bars: 1.0 mm.

Figs 7A–C , 13D , 17D

Lycocerus putzi Švihla, 2011: 11, figs 12, 60, 61–63.

##### Non-type material examined.

China: 1♂1♀(NHMB), Sichuan, Chengdu, Qingchengshan, 1360 m, 30°44'N, 103°08'E (DDM), 28.VIII.2004, leg. S. Murzin; 1♂ (IZAS), Sichuan, Emeishan, Qingyinge, 800–1000 m, 25.IV.1957, lg. K. R. Huang.

##### Descriptive notes.

**Male.** Aedeagus: basal piece feebly longer than dorsal plate of each paramere (Fig. 7A–C); ventral process of each paramere nearly straight and approaching to each other in ventral view (Fig. 7A), nearly vertical in lateral view (Fig. 7C); dorsal plates of parameres obviously longer than ventral processes (Fig. 7A, C), outer margins obviously converging apically, apical margins rounded in dorsal view (Fig. 7B); laterophyse feebly longer than ventral process, with apices acute and appreciably directing dorso-outwards (Fig. 7A, C).

**Female**. Internal organ of reproductive system (Fig. 13D): spermatheca obviously longer than diverticulum; accessory gland ~ 2.5× longer than spermatheca.

Abdominal sternite VIII (Fig. 17D): triangular emargination in middle and subrounded emarginations on both sides of posterior margin, lateral emarginations feebly deeper and obviously wider than the middle one, the portion between lateral and middle emarginations narrow and acute at apices, obviously extending over apices of latero-apical angles, which are rounded.

##### Distribution.

China (Sichuan).

##### Remarks.

The aedeagus of this species was illustrated only for apical parts by Švihla (2011). Here, the aedeagus is illustrated with general views, and the female internal genitalia is illustrated for the first time.

#### ﻿ Lycocerus pictipennis

Taxon classificationAnimaliaColeopteraCantharidae

(Wittmer, 1995)

C8CD4A7A-86C4-53C0-8B8C-7E301471D731

Fig. 7D–F

Athemus (Isathemus) pictipennis Wittmer, 1995: 276, figs 142, 143.

##### Type material examined.

***Holotype***: 1♂ (NHMB), China, Yunnan, Dongchuan, 26°31'07'N, 103°14'E (DDM), 28.VI–3.VII.1994, leg. Vit Kubáň.

##### Non-type material examined.

1♂ (MHBU), Myanmar, Prov. Kachin Mt Emaw Bum, 2358 m road of Kanphant, 26°09'N, 98°31'E (DDM), 28.V.2006, leg. L. Langer.

##### Descriptive notes.

**Male.** Aedeagus: basal piece as long as dorsal plate of each paramere (Fig. 7D–F); ventral process of each paramere thin and bent inwards in ventral view (Fig. 7D), inclining ventrally in lateral view (Fig. 7F); dorsal plates of parameres obviously longer than ventral processes (Fig. 7D, F), with apical margins rounded in lateral view (Fig. 7E); laterophyse slightly shorter than ventral process, with apices acute and directing dorso-outwards (Fig. 7D, F).

##### Distribution.

China (Yunnan); Myanmar (new record).

##### Remarks.

This species was missing in the Palaearctic Catalogue by Kazantsev and Brancucci (2007). It is recorded to Myanmar for the first time herein. The aedeagus of this species was illustrated only in ventral and lateral views by Wittmer (1995). Here, the aedeagus is illustrated in ventral, dorsal, and lateral views, and the abdominal sternite VIII and internal genitalia of female are illustrated for the first time.

#### ﻿ Lycocerus curvatus

Taxon classificationAnimaliaColeopteraCantharidae

(Wittmer, 1995)

58967A46-725B-54ED-ACAE-EC42117ABA11

Fig. 11A–C

Athemus (Isathemus) curvatus Wittmer, 1995: 260, figs 117, 118, 188.
Lycocerus curvatus : Kazantsev and Brancucci 2007: 250.

##### Type material examined.

***Holotype***: 1♂ (NHMB), China, Sichuan, Liziping, 28.VI–3.VII.1991, lgt. R. Dunda.

##### Non-type material examined.

China: 1♂ (IZAS), Sichuan, Emeishan, 2100 m, 25.VI.1955, leg. X. K Bu; 1♂ (IZAS), same locality as the preceding, 2100–3100 m, 25.VI.1955, leg. X. C. Yang.

##### Descriptive notes.

**Male.** Aedeagus: basal piece nearly as long as dorsal plate of each paramere (Fig. 11A–C); ventral process of each paramere slender and bent inwards apically in ventral view (Fig. 11A), inclining ventrally in lateral view (Fig. 11C); dorsal plates of parameres feebly longer than ventral processes (Fig. 11A, C), with apical margins rounded in dorsal view (Fig. 11B); laterophyse slightly shorter than ventral process, with apices acute and appreciably directing dorso-outwards (Fig. 11A, C).

##### Distribution.

China (Sichuan).

##### Remarks.

The aedeagus of this species was illustrated only in ventral and lateral views by Wittmer (1995). Here, the aedeagus is illustrated in ventral, dorsal, and lateral views, and the female internal genitalia is illustrated for the first time.

#### ﻿ Lycocerus laterophysus

Taxon classificationAnimaliaColeopteraCantharidae

Y. Yang, Wang & Liu sp. nov.

6D2DE3CD-53AE-5D54-BB99-0984B6599674

https://zoobank.org/D433A884-731B-41D2-ABA2-3F813CF640C1

**Figure 8.**
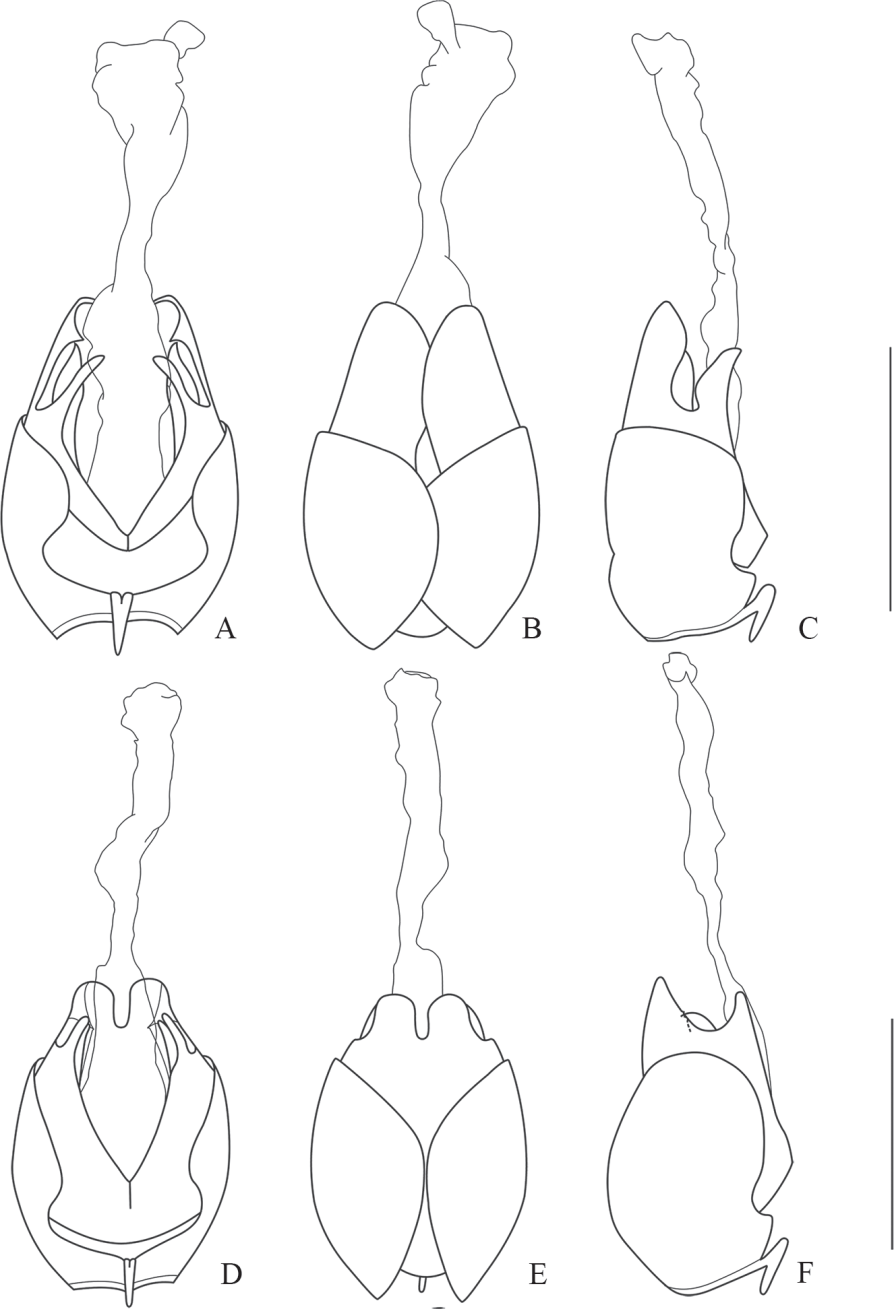
Aedeagus **A–C***Lycoceruslaterophysus* sp. nov. **D–F***L.flavipennis* sp. nov. **A, D** ventral view **B, E** dorsal view **C, F** lateral view. Scale bars: 1.0 mm.

Figs 8A–C , 14D , 16H , 18A, B

##### Type material.

***Holotype***: ♂ (MHBU), China, Guangxi, Wuming, Damingshan, 1230–1423 m, 20.V.2011, leg. H. Y. Liu. ***Paratypes***: China: 4♀1♂ (MHBU), same data as holotype; 2♀ (MHBU), same locality as holotype, 1100 m, 27.V.2011, leg. H. Y. Liu; 1♀ (MHBU), same locality as holotype, 600–900 m, 25.V.2011, leg. H. Y. Liu.

##### Diagnosis.

The new species can be easily distinguished from all others by its body coloration, head and pronotum bicolored, with vertex black and clypeus yellow, pronotum with a black wide longitudinal median band (Fig. 18A, B). Also, its aedeagus is unique and differs from all others in the ventral process of each paramere expanded near base in lateral view (Fig. 8C), and broad laterophyse in ventral view (Fig. 8A).

##### Description.

**Male** (Fig. 18A). Head, prothorax and legs orange, vertex black, mandibles dark brown, antennomeres III–XI black, pronotum with a wide black longitudinal median band extending from posterior to anterior margins, scutellum black, elytra pale yellow, tarsi darkened, meso- and metasterna and abdomen black. Body densely covered with yellow recumbent pubescence.

Head feebly narrowed behind eyes, surface densely and finely punctate; eyes moderately large and protruding, head width across eyes feebly wider than anterior margin of pronotum; antennae filiform, extending to elytral mid-length when reclined, antennomere II shortest, ~ 2.5× longer than wide at apex, III–V feebly widened apically, IV–X each with a short smooth impression near apical part of outer margin, V longest, VI–XI nearly parallel-sided, XI acute at apex.

Pronotum distinctly longer than wide, anterior margin feebly arcuate, lateral margins subparallel, posterior margin nearly straight, anterior angles rounded, posterior angles right-angled, disc convex on posterolateral parts, surface finely and feebly sparsely punctate than that on head.

Elytra ~ 4.5× longer than pronotum, 5.4× longer than width across humeri, outer margins nearly parallel, disc semi-lustrous, coarsely and densely punctate.

Legs slender, all tarsal claws simple.

Aedeagus: basal piece nearly as long as dorsal plate of each paramere (Fig. 8A–C); ventral process of each paramere slender, feebly bent inwards and approaching each other in ventral view (Fig. 8A), slightly expanded near base and bent ventrally in lateral view (Fig. 8C); dorsal plates of parameres obviously longer than ventral process (Fig. 8A, C), with apical margins arcuate and descending inwards in dorsal view (Fig. 8B); laterophyse nearly as long as ventral process and broad in ventral view, with apices acute and directing dorso-outwards (Fig. 8A, C).

**Female** (Fig. 18B). Similar to the males, but eyes less protruding, antennae thinner and shorter, extending to basal one-third length of elytra when reclined, middle antennomeres without impressions, fore and middle legs with a digitiform tooth on each anterior claw, pronotum feebly longer than wide.

Internal organ of reproductive system (Fig. 14D): spermatheca feebly shorter than diverticulum; accessory gland ~ 2.5× longer than spermatheca.

Abdominal sternite VIII (Fig. 16H): hardly emarginate in middle and subtriangular emarginations on both sides of posterior margin, lateral emarginations obviously deeper than the middle one, the portion between lateral and middle emarginations wide and rounded at apices, obviously extending over apices of latero-apical angles, which are subrounded.

Body length: 9.3–9.7 mm (9.3 mm in holotype); width: 1.8–2.0 mm (1.8 mm in holotype).

##### Distribution.

China (Guangxi).

##### Etymology.

The new species is named after the quite wide laterophyse of its aedeagus.

#### ﻿ Lycocerus flavipennis

Taxon classificationAnimaliaColeopteraCantharidae

Y. Yang, Wang & Liu sp. nov.

ED2F438C-8674-594F-A98D-7039E0173457

https://zoobank.org/A565BCEE-4AF7-454A-8F7E-D0650A58A1FB

Figs 8D–F , 14A , 16E , 18C, D

##### Type material.

***Holotype***: ♂ (MHBU), China, Zhejiang, Lin’an, Qingliangfeng, 16–22.V.2012, leg. G. L. Xie. ***Paratypes***: China: 2♀ (MHBU), same data as holotype; 1♀ (MHBU), Zhejiang, Lin’an, Qingliangfeng, Shunxiwu, 15–18.V.2012, leg. J. S. Xu & L. X. Chang; 1♀ (MHBU), Zhejiang, Lin’an, Qingliangfeng, Longtangshan, 19.V.2011, leg. G. L. Xie.

##### Diagnosis.

The new species resembles *L.pictipennis* in the coloration and characteristics of tarsal claws, but differs from the latter in the aedeagus: basal piece very large, ~ 3× longer than dorsal plate of each paramere in lateral view (Fig. 8F), dorsal plate narrow (Fig. 8E), laterophyse slightly bent inwards in ventral view (Fig. 8D). In comparison, in the aedeagus of *L.pictipennis*, basal piece is nearly as long as dorsal plate of each paramere in lateral view (Fig. 7F), dorsal plate wide (Fig. 7E), laterophyse obviously bent outwards in ventral view (Fig. 7D).

Also, it is similar to *L.kubani* in the structure of tarsal claws, genitalia of both sexes and female abdominal sternite VIII, but which are different in each component part in detail, and can be easily distinguished from the latter in the uniformly yellow elytra, while elytra black at apices in *L.kubani*. The coloration of elytra is stable and a good character to recognize the species in *L.pallidulus* group.

##### Description.

**Male** (Fig. 18C). Head, prothorax and legs yellowish orange, mandibles dark brown at apices, antennomeres III–XI black, scutellum orange and darkened along margins, elytra pale yellow, tibiae and tarsi black, except for protibiae yellow ventrally, meso- and metasterna and abdomen yellowish brown. Body densely covered with yellow recumbent pubescence.

Head feebly narrowed behind eyes, surface densely and finely punctate; eyes moderately large and protruding, head width across eyes distinctly wider than anterior margin of pronotum; antennae filiform, extending to elytral mid-length when reclined, antennomere II shortest, ~ 2× longer than wide at apex, III–IX feebly widened apically, IV–IX each with a short smooth impression near basal part of outer margin (X–XI missing), VII longest.

Pronotum distinctly longer than wide, anterior margin feebly arcuate, lateral margins subparallel, posterior margin nearly straight, anterior angles rounded, posterior angles right-angled, disc convex on posterolateral parts, surface finely and feebly sparsely punctate than that on head.

Elytra ~ 3.5× longer than pronotum, 4.4× longer than width across humeri, outer margins nearly parallel, disc semi-lustrous, coarsely and densely punctate.

Legs slender, fore and middle legs with a digitiform tooth on each anterior and posterior claw, and hind claws simple.

Aedeagus: basal piece obviously longer than dorsal plate of each paramere (Fig. 8D–F); ventral process of each paramere thin and short, feebly bent inwards and approaching to each other in ventral view (Fig. 8D), nearly straight in lateral view (Fig. 8F); dorsal plates of parameres obviously longer than ventral processes (Fig. 8D, F), with inner margins parallel, outer margins abruptly converging apically in the middle, apical margins rounded (Fig. 8E); laterophyse feebly shorter than ventral process, bent dorsally, with apices acute and directing dorso-inwards in ventral view (Fig. 8D, F).

**Female** (Fig. 18D). Similar to the males, but eyes less protruding, antennae thinner and shorter, extending to basal one-third length of elytra when reclined, middle antennomeres without impressions, pronotum nearly as long as wide.

Internal organ of reproductive system (Fig. 14A): spermatheca slightly longer than diverticulum; accessory gland ~ 1.5× longer than spermatheca.

Abdominal sternite VIII (Fig. 16E): triangular emargination in middle and rounded emarginations on both sides of posterior margin, lateral emarginations wider and feebly deeper than the middle one, the portions between lateral and middle emarginations narrow and acute at apices, obviously extending over apices of latero-apical angles, which are widely triangular.

Body length: 11.0–12.0 mm (11.0 mm in holotype); width: 2.3–3.0 mm (2.3 mm in holotype).

##### Distribution.

China (Zhejiang).

##### Etymology.

The specific name is derived from the Latin *flavus* (golden-yellow) and *pinna* (wing), referring to its yellow elytra.

#### ﻿ Lycocerus maoershanensis

Taxon classificationAnimaliaColeopteraCantharidae

Y. Yang, Liu & X. Yang sp. nov.

245723F0-2023-5829-B45E-05247F233231

https://zoobank.org/482BA762-7D56-4D21-9EA8-FF1CAD1998C7

**Figure 9.**
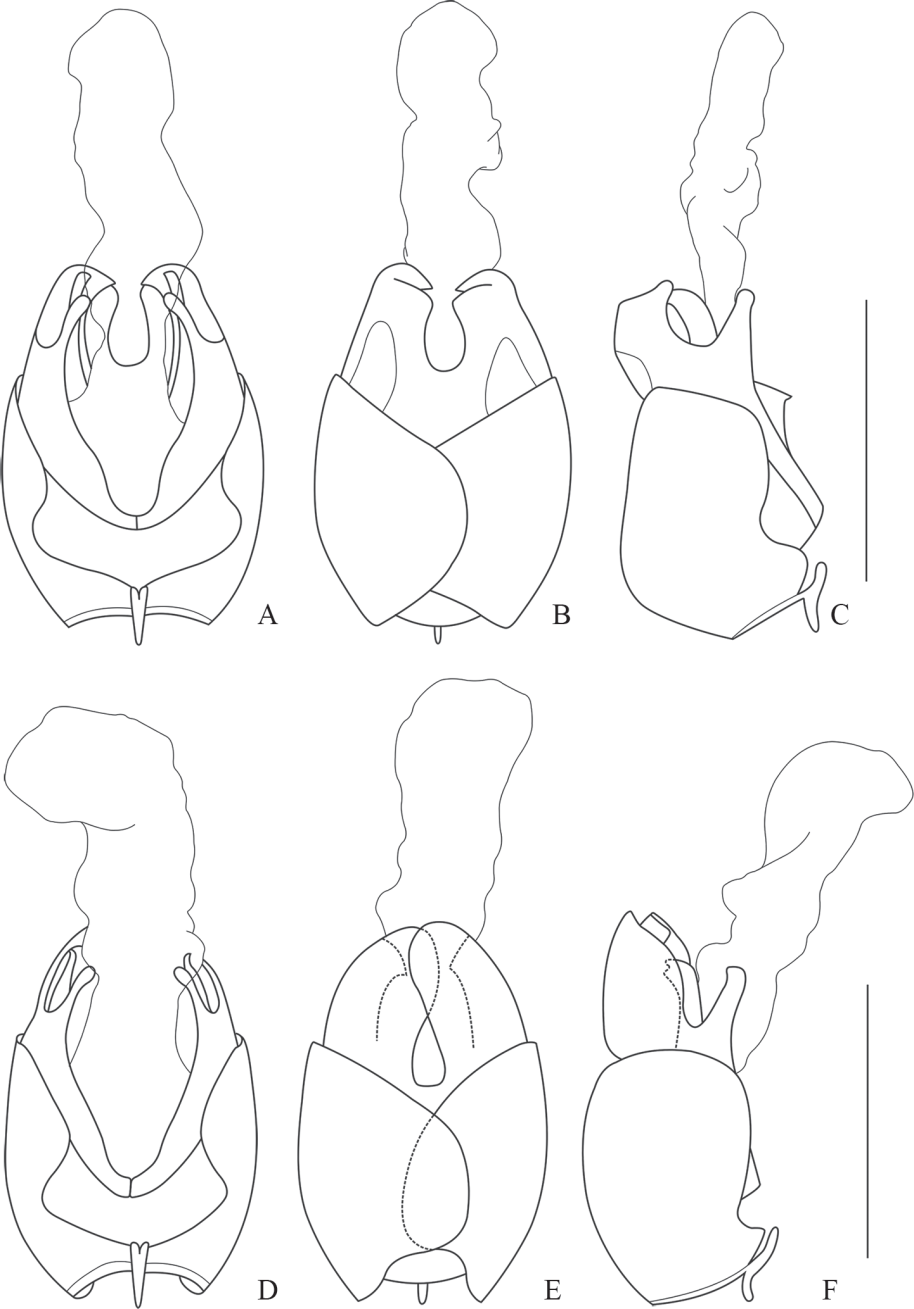
Aedeagus **A–C***Lycocerusmaoershanensis* sp. nov. **D–F***L.putzimimus* sp. nov. **A, D** ventral view **B, E** dorsal view **C, F** lateral view. Scale bars: 1.0 mm.

Figs 9A–C , 13C , 17C , 19A, B

##### Type material.

***Holotype***: ♂ (IZAS), China, Guangxi, Maoershan, 1900 m, 14.VII.1985, leg. S. B. Liao. ***Paratypes***: China: 1♂, 1♀ (IZAS), same data as holotype; 1♀ (IZAS), same locality as holotype, 1950 m, 14.VII.1985, leg. S. M. Song.

##### Diagnosis.

Although the new species is similar to *L.laterophysus* sp. nov. in the bicolored head, it is more related to *L.zdeneki* on basis of the structure of aedeagus. Further, *L.maoershanensis* sp. nov. can be distinguished from the latter in the following characters: scutellum dark brown; female abdominal sternite VIII (Fig. 17C) with the portion between lateral and middle emarginations rounded at apices, which obviously extending over latero-apical angles. In comparison, *L.zdeneki* has yellow scutellum; female abdominal sternite VIII (Fig. 16F) with the portion between lateral and middle emarginations feebly extending over latero-apical angles.

##### Description.

**Male** (Fig. 19A). Head, prothorax and legs yellowish orange, vertex with a small triangular dark brown marking, mandibles dark brown at apices, antennomeres III–XI black, pronotum with two dark brown irregular markings near middle of anterior and posterior margins, scutellum black, elytra pale yellow and almost transparent, legs more or less darkened at tarsi, apices of tibiae and femora, meso- and metasterna and abdomen black. Body densely covered with yellow recumbent pubescence.

Head feebly narrowed behind eyes, surface densely and finely punctate; eyes moderately large and protruding, head width across eyes slightly wider than anterior margin of pronotum; antennae filiform, extending to three-fifths of elytra when reclined, antennomere II shortest, ~ 1.5× longer than wide at apex, III–VI feebly widened apically, IV–XI each with a short smooth impression near basal part of outer margin, VII–XI nearly parallel-sided, VIII longest.

Pronotum subquadrate, slightly longer than wide, anterior margin feebly arcuate, lateral margins subparallel, posterior margin nearly straight, anterior angles rounded, posterior angles obtuse-angled, disc convex on posterolateral parts, surface finely and feebly sparsely punctate than that on head.

Elytra ~ 3.75× longer than pronotum, 5.0× longer than width across humeri, outer margins nearly parallel, disc semi-lustrous, coarsely and densely punctate.

Legs slender, fore and middle legs with a digitiform tooth on each anterior claw, and hind claws simple.

Aedeagus: basal piece slightly longer than dorsal plate of each paramere (Fig. 9A–C); ventral process of each paramere slender, feebly bent inwards and approaching to each other in ventral view (Fig. 9A), inclining ventrally in lateral view (Fig. 9C); dorsal plates of parameres slightly longer than ventral processes (Fig. 9A, C), with inner margins emarginate at apical parts, outer margins slightly converging apically, apical margins slightly arcuate and descending inwards, inner apical angle acute angled and outer angle rounded in dorsal view (Fig. 9B); laterophyse feebly longer than ventral process, with apices acute and directing dorso-inwards in ventral view (Fig. 9A).

**Female** (Fig. 19B). Similar to the males, but eyes less protruding, antennae shorter and extending to basal third length of elytra when reclined, middle antennomeres without impressions, pronotum nearly as long as wide, fore and middle legs with a digitiform tooth on each anterior and posterior claw.

Internal organ of reproductive system (Fig. 13C): spermatheca nearly as long as diverticulum; accessory gland nearly as long as spermatheca.

Abdominal sternite VIII (Fig. 17C): subrounded emarginations in middle and on both sides of posterior margin, lateral emarginations deeper than and as wide as the middle one, the portions between lateral and middle emarginations moderately wide and rounded at apices, obviously extending over apices of latero-apical angles, which are narrowly triangular.

Body length: 9.0–10.0 mm (9.1 mm in holotype); width: 1.7–2.3 mm (1.8 mm in holotype).

##### Distribution.

China (Guangxi).

##### Etymology.

The specific name is derived from the name of the type locality, Maoershan, Guangxi, China.

#### ﻿ Lycocerus putzimimus

Taxon classificationAnimaliaColeopteraCantharidae

Y. Yang, Wang & Liu sp. nov.

B1C0074D-D9F8-5CBF-B09E-7F164E866346

https://zoobank.org/C42DA1C1-F720-470F-BEB2-C0DA470121CE

Figs 9D–F , 19C

##### Type material.

***Holotype***: ♂ (MHBU), China, Yunnan, Pingbian, Daweishan, 1900 m, 24.V.1996, leg. L. Y. Zheng.

##### Diagnosis.

The new species seems similar to *L.putzi* in the coloration, but differs in the following characters: fore and mid legs with a digitiform tooth on each anterior claw in male, while on both anterior and posterior claws in the latter; aedeagus with dorsal plate of each paramere wide (Fig. 9E) and moderately longer than ventral process (Fig. 9F), while narrower (Fig. 7B) and distinctly long (Fig. 7C) in *L.putzi*; laterophyse feebly furcate at apices (Fig. 9F), while acute in the latter (Fig. 7C).

##### Description.

**Male** (Fig. 19C). Head, prothorax and legs orange, mandibles dark brown at apices, antennae black, except for bases of antennomeres I yellow, pronotum with a small and round black marking in center of disc, scutellum yellow, elytra pale yellow and almost transparent, each with a black longitudinal band along apical two-thirds part, leaving a long triangular area pale yellow along suture, legs darkened at tarsi, meso- and metasterna and abdomen black, terminal two abdominal ventrites and sternites pale yellow. Body densely covered with yellow recumbent pubescence.

Head feebly narrowed behind eyes, surface densely and finely punctate; eyes moderately large and protruding, head width across eyes slightly wider than anterior margin of pronotum; antennae filiform, extending to apical third length of elytra when reclined, antennomeres II shortest, ~ 2× longer than wide at apices, IV–XI nearly parallel-sided, each with a short smooth impression near apical part of outer margin, IV longest.

Pronotum subquadrate, feebly longer than wide, anterior margin feebly arcuate, lateral margins subparallel, posterior margin nearly straight, anterior angles obtuse-rounded, posterior angles nearly right-angled, disc convex on posterolateral parts, surface finely and feebly sparsely punctate than that on head.

Elytra ~ 4.4× longer than pronotum, 3.12× longer than width across humeri, outer margins nearly parallel, disc semi-lustrous, coarsely and densely punctate.

Legs slender, fore and middle legs with a digitiform tooth on each anterior claw, and hind claws simple.

Aedeagus: basal piece slightly longer than dorsal plate of each paramere (Fig. 9D–F); ventral process of each paramere slender, slightly expanded at apices, slightly bent inwards and approaching to each other in ventral view (Fig. 9D), inclining ventrally in lateral view, with apices slightly curved dorsally (Fig. 9F); dorsal plate obviously longer than ventral process of each paramere, with apical margins rounded (Fig. 9E); laterophyse feebly longer than ventral process, with apices bifurcate and directing dorso-outwards (Fig. 9D, F).

**Female.** Unknown.

Body length: 10.2 mm; width: 2.2 mm.

##### Distribution.

China (Yunnan).

##### Etymology.

The specific name is derived from the Latin *mimus* (imitator), referring to its similarity to *L.putzi* Švihla, 2011.

#### ﻿ Lycocerus chongqingensis

Taxon classificationAnimaliaColeopteraCantharidae

Y. Yang, Wang & Liu sp. nov.

BD56996A-F1E8-5887-9A29-10FDE46A71E0

https://zoobank.org/74057CE9-07BD-4C2E-9F51-55584FDCDC9D

**Figure 10.**
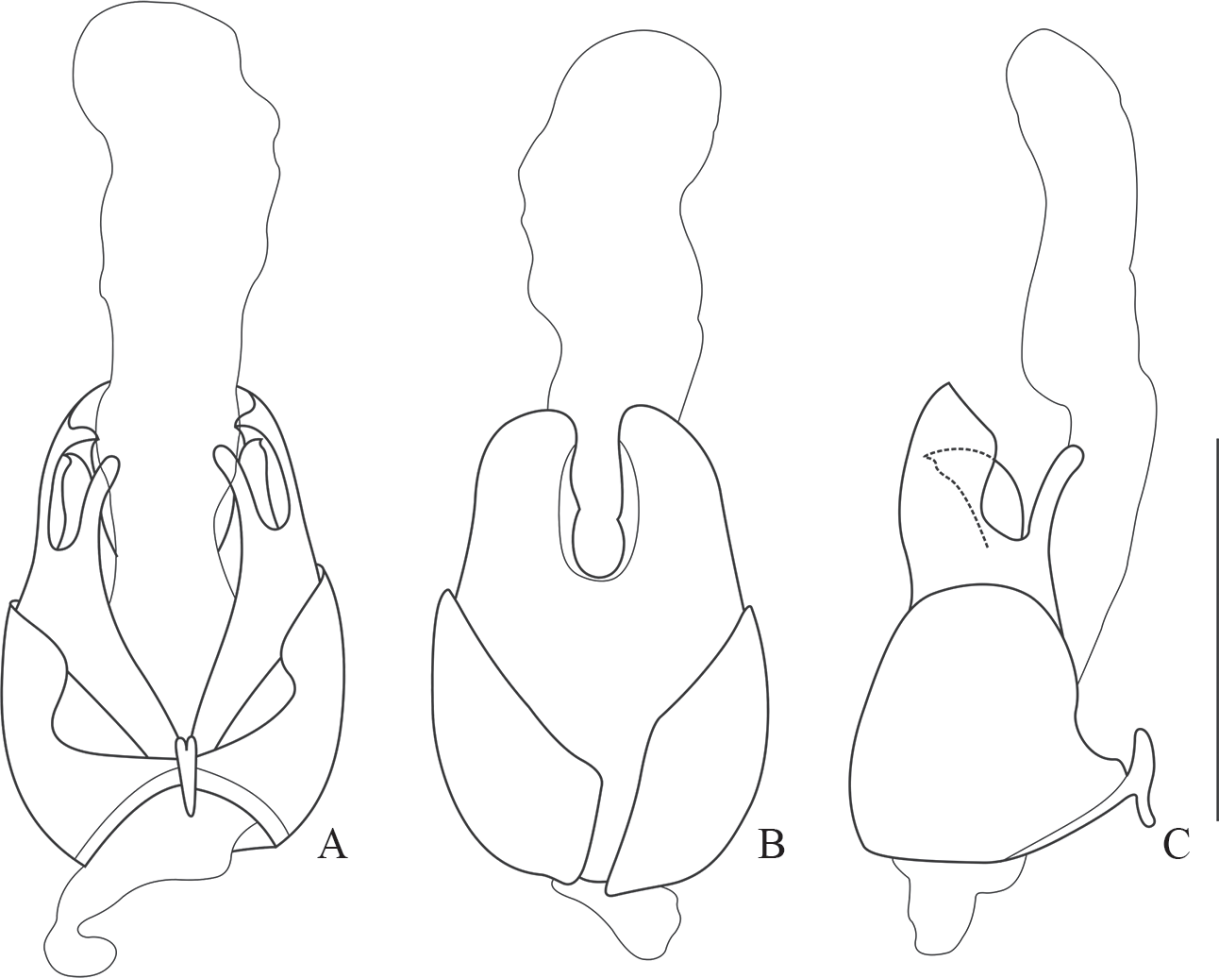
Aedeagus of *Lycoceruschongqingensis* sp. nov. **A** ventral view **B** dorsal view **C** lateral view. Scale bars: 1.0 mm.

Figs 10A–C , 15A , 17E , 20A, B

##### Type material.

***Holotype***: ♂ (MHBU), China, Chongqing, Nanchuan, Jinfoshan, 23–24.VII.2003, leg. Y. S. Liu & C. X. Yuan. ***Paratypes***: 1♂2♀ (MHBU), same data as holotype.

##### Diagnosis.

The new species seems most similar to *L.centrochinensis* in both coloration and characteristics of tarsal claws, but differs in the following characters: body size is smaller; aedeagus: dorsal plates of parameres with inner margins feebly protuberant near base (Fig. 10B); abdominal sternite VIII (Fig. 17E) moderately narrowed posteriorly, the portion between lateral and middle emarginations rounded at apices, which slightly extending over apices of latero-apical angles. In comparison, *L.centrochinensis* has larger body; aedeagus: dorsal plates of parameres with inner margins abruptly diverging near middle (Fig. 3E); abdominal sternite VIII (Fig. 16B) strongly narrowed posteriorly, the portion between lateral and middle emarginations triangular at apices, which distinctly extending over apices of latero-apical angles.

##### Description.

**Male** (Fig. 20A). Head, prothorax, scutellum and legs orange, mandibles dark brown at apices, antennomeres III–XI black, elytra pale yellow, legs darkened at tarsi, meso- and metasterna and abdomen black. Body densely covered with pale yellow recumbent pubescence.

Head feebly narrowed behind eyes, surface densely and finely punctate; eyes moderately large and protruding, head width across eyes wider than anterior margin of pronotum; antennae filiform, extending to apical third of elytra when reclined, antennomere II shortest, ~ 2× longer than wide at apex, III–IV feebly expanded at apex, IV–X each with a short smooth impression near apical part of outer margin, V–XI nearly parallel-sided, VIII longest.

Pronotum subquadrate, feebly longer than wide, anterior margin feebly arcuate, lateral margins subparallel, posterior margin nearly straight, anterior angles obtuse-rounded, posterior angles nearly right-angled, disc convex on posterolateral parts, surface finely and feebly sparsely punctate than that on head.

Elytra ~ 4.6× longer than pronotum, 3.3× longer than width across humeri, outer margins nearly parallel, disc semi-lustrous, coarsely and densely punctate.

Legs slender, all claws simple.

Aedeagus: basal piece nearly as long as dorsal plate of each paramere (Fig. 10A–C); ventral process of each paramere slender and feebly bent inwards and approaching to each other in ventral view (Fig. 10A), inclining ventrally in lateral view (Fig. 10C); dorsal plates of parameres obviously longer than ventral processes (Fig. 10A, C), with inner margins nearly parallel, feebly triangularly protuberant near base, outer margins slightly converging apically, apical margins slightly descending outwards, inner and outer apical angles rounded in dorsal view (Fig. 10B); laterophyse nearly as long as ventral process, with apices acute and directing dorso-outwards (Fig. 10A, C).

**Female** (Fig. 20B). Similar to the males, but eyes less protruding, antennae shorter, not reaching elytral mid-length when reclined, middle antennomeres without impressions, pronotum nearly as long as wide, fore and middle legs with a digitiform tooth on each anterior and posterior claw.

Internal organ of reproductive system (Fig. 15A): spermatheca nearly as long as diverticulum; accessory gland ~ 2.2× longer than spermatheca.

Abdominal sternite VIII (Fig. 17E): hardly emarginate in middle and subrounded emarginations on both sides of posterior margin, lateral emarginations obviously deeper than the middle one, the portion between lateral and middle emarginations wide and rounded at apices, slightly extending over apices of latero-apical angles, which are truncated.

Body length: 7.8–9.0 mm (8.0 mm in holotype); width: 1.7–2.1 mm (1.8 mm in holotype).

##### Distribution.

China (Chongqing).

##### Etymology.

The specific name is derived from the type locality, Chongqing, China.

#### ﻿ Lycocerus bispermathecus

Taxon classificationAnimaliaColeopteraCantharidae

Y. Yang, Wang & Liu sp. nov.

868F1287-D4E8-54AD-AF8F-895BE818FA24

https://zoobank.org/372D5287-5C6F-484C-8EAC-C8CC9DD0972E

**Figure 11.**
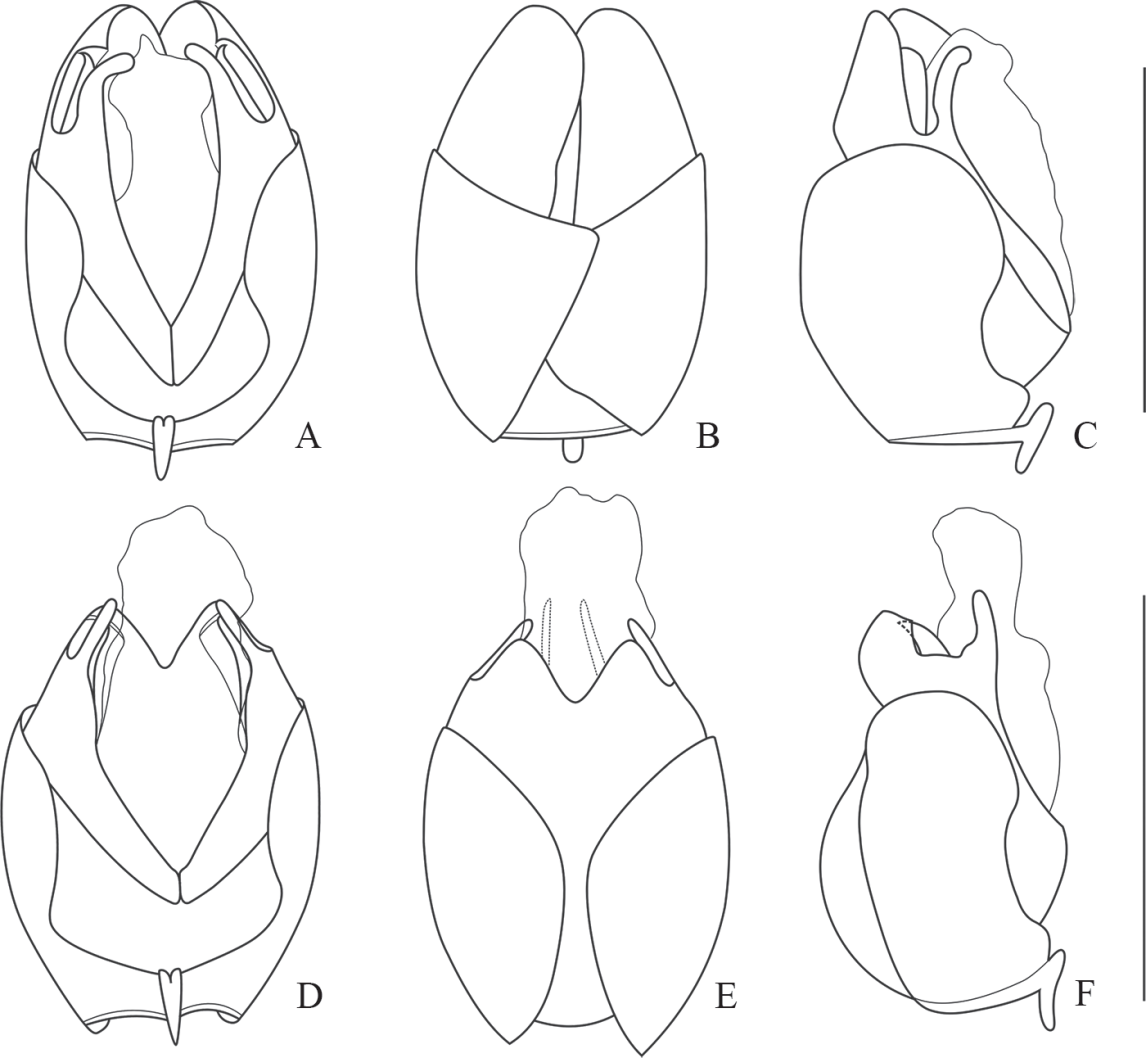
Aedeagus **A–C***Lycoceruscurvatus* (Wittmer, 1995) **D–F***L.bispermathecus* sp. nov. **A, D** ventral view **B, E** dorsal view **C, F** lateral view. Scale bars: 1.0 mm.

Figs 11D–F , 15B , 17F , 20C, D

Lycocerus centrochinensis (Švihla, 2004): Li et al. 2015: 300, fig. 1A [misidentification].

##### Type material.

***Holotype***: ♂ (MHBU), China, Ningxia, Kongtongshan, 6.VI.1992, leg. J. L. Ding. ***Paratypes***: China: 1♀ (MHBU), Ningxia, Jingyuan, Liupanshan, 13.VI.1995, Collectors Group III of Forestry; 1♀ (MHBU), same data as the preceding, 8.VI.1995, Collectors Group III of Forestry; 1♀ (MHBU), same data as the preceding, 17.VI.1995, Collectors Group III of Forestry.

##### Diagnosis.

The new species seems similar to *L.hubeiensis* in the coloration, but differs in the following characters: tarsal claws simple in males, while fore and mid-anterior and posterior claws each with a digitiform tooth at base in the latter; aedeagus: dorsal plates of parameres triangular at apices (Fig. 11E), while truncated in the latter (Fig. 4E); spermatheca with two spiral tubes (Fig. 15B), while only one in the latter.

**Figure 12.**
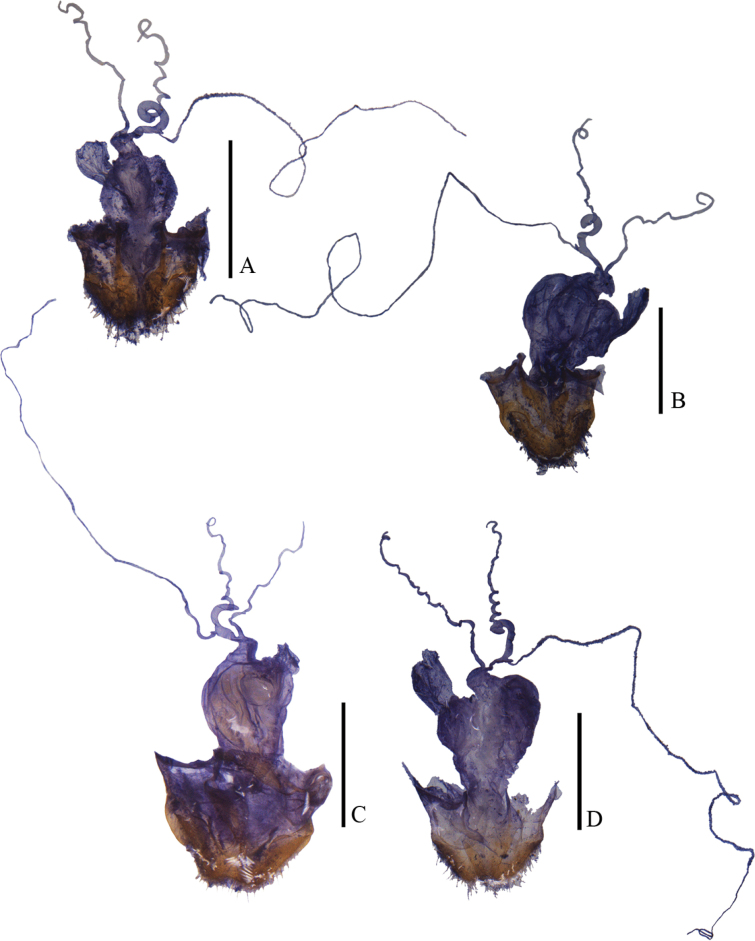
Internal organ of female reproductive system, lateral view **A***Lycocerushubeiensis* (Švihla, 2004) **B***L.centrochinensis* (Švihla, 2004) **C***L.guerryi* (Pic, 1906) **D***L.genaemaculatus* (Wittmer, 1951). Scale bars: 1.0 mm.

##### Description.

**Male** (Fig. 20C). Head, prothorax, scutellum and legs yellowish orange, mandibles dark brown at apices, antennomeres III–XI black, elytra pale yellow and almost transparent, black at apices, legs darkened at tarsi, meso- and metasterna and abdomen yellowish brown. Body densely covered with yellow recumbent pubescence.

Head feebly narrowed behind eyes, surface densely and finely punctate; eyes moderately large and protruding, head width across eyes nearly wider than anterior margin of pronotum; antennae filiform, extending to apical third length of elytra when reclined, antennomere II shortest, ~ 2× longer than wide at apices, IV–XI nearly parallel-sided, each with a short smooth impression near apical part of outer margin, IV longest.

Pronotum subquadrate, slightly longer than wide, anterior margin feebly arcuate, lateral margins subparallel, posterior margin nearly straight, anterior angles obtuse-rounded, posterior angles nearly right-angled, disc convex on posterolateral parts, surface finely and feebly sparsely punctate than that on head.

Elytra ~ 4.4× longer than pronotum, 3.12× longer than width across humeri, outer margins nearly parallel, disc semi-lustrous, coarsely and densely punctate.

Legs slender, all claws simple.

Aedeagus: basal piece obviously longer than dorsal plate of each paramere (Fig. 11D–F); ventral process of each paramere slender and straight, approaching to each other in ventral view (Fig. 11D) and feebly inclining dorsally in lateral view (Fig. 11F); dorsal plates of parameres feebly shorter than ventral processes (Fig. 11D, F), with inner margins diverging and outer margins converging apically, apical margins widely triangular in dorsal view (Fig. 11E); laterophyse obviously shorter than ventral process, with apices acutely hooked, directing dorso-inwards in ventral view (Fig. 11D); inner sac with a pair of longitudinal sclerites on dorsal side (Fig. 11E).

**Figure 13.**
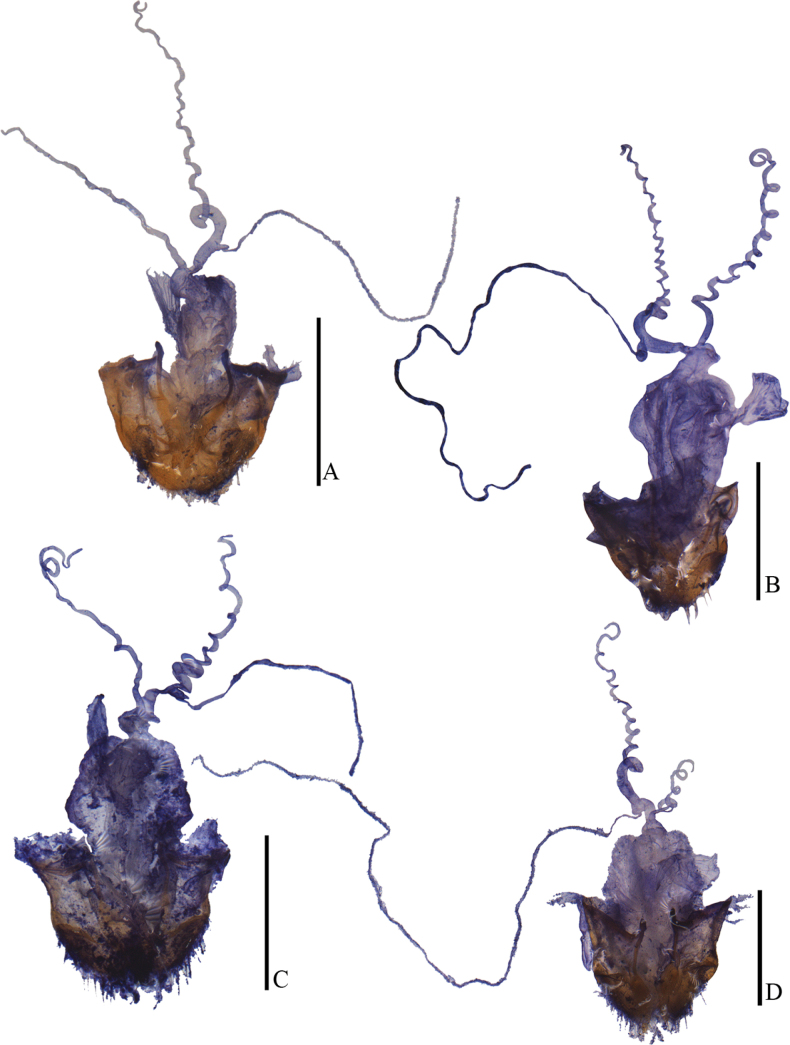
Internal organ of female reproductive system, lateral view **A***Lycocerusbilineatus* (Wittmer, 1995) **B***L.jelineki* (Švihla, 2004) **C***L.maoershanensis* sp. nov. **D***L.putzi* Švihla, 2011. Scale bars: 1.0 mm.

**Figure 14.**
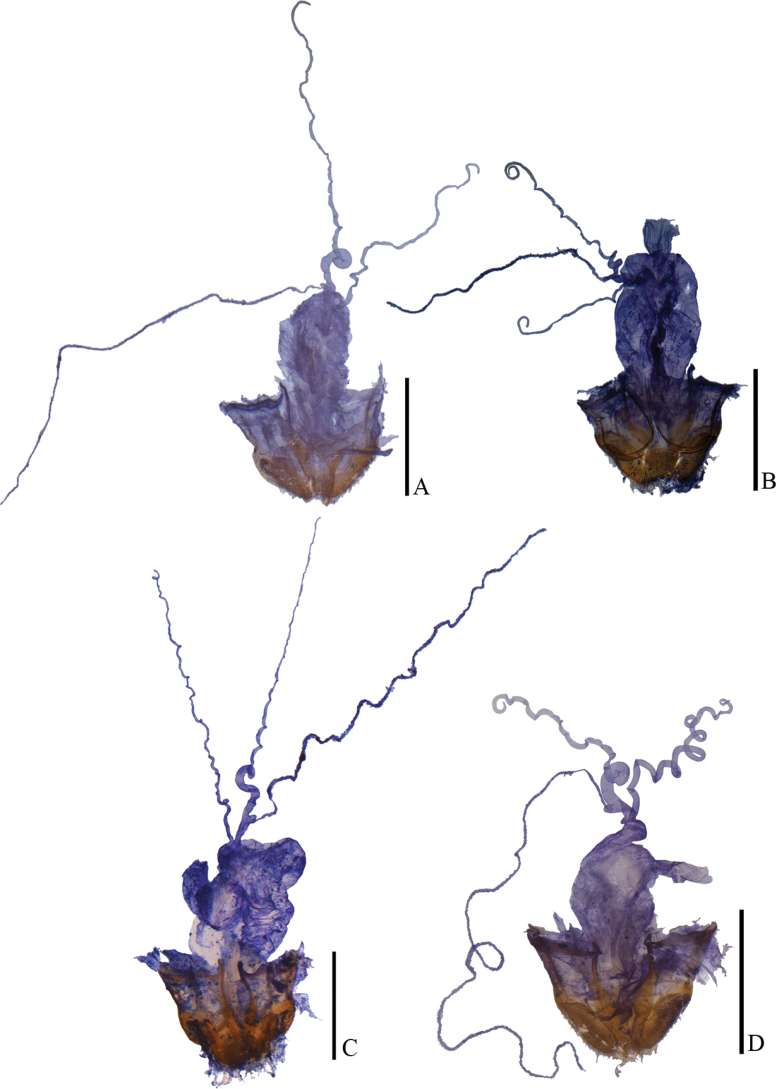
Internal organ of female reproductive system, lateral view **A***L.flavipennis* sp. nov. **B***L.zdeneki* (Švihla, 2004) **C***L.kubani* (Švihla, 2004) **D***L.laterophysus* sp. nov. Scale bars: 1.0 mm.

**Female** (Fig. 20D). Similar to the males, but eyes less protruding, antennae shorter and extending to elytral mid-length when reclined, middle antennomeres without impressions, pronotum nearly as long as wide, fore and middle legs with a digitiform tooth on each anterior and posterior claw.

**Figure 15.**
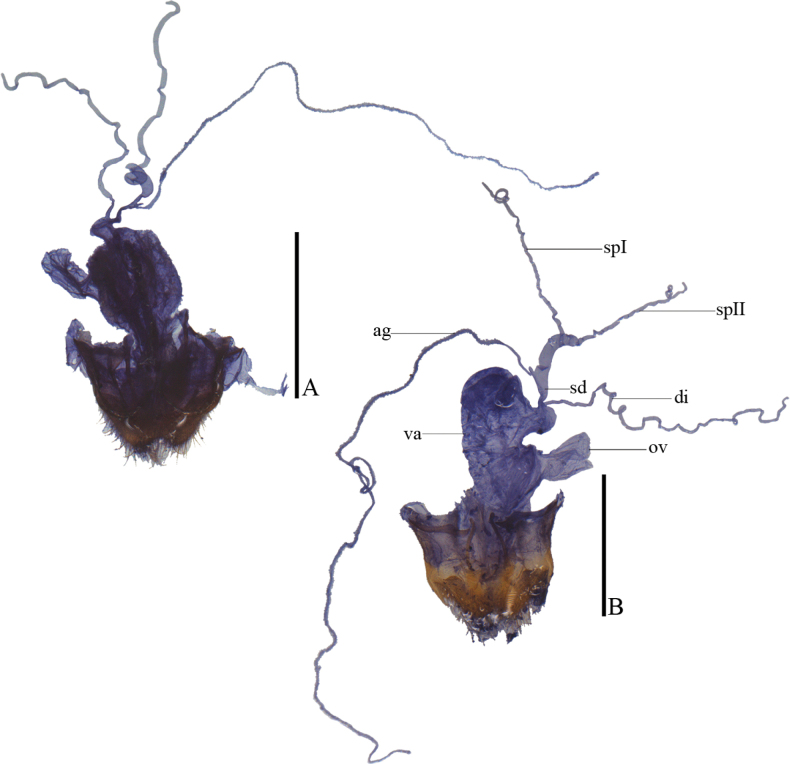
Internal organ of female reproductive system, lateral view **A***L.chongqingensis* sp. nov. **B***L.bispermathecus* sp. nov. Abbreviations: ag – accessory gland; di – diverticulum; sd – spermathecal duct; sp – spermatheca; ov – median oviduct; va – vagina. Scale bars: 1.0 mm.

Internal organ of reproductive system (Fig. 15B): spermathecal duct stout, spermatheca with two spiral tubes, which are subequal in length, both of them shorter than diverticulum; accessory gland ~ 2.5× longer than spermatheca.

**Figure 16.**
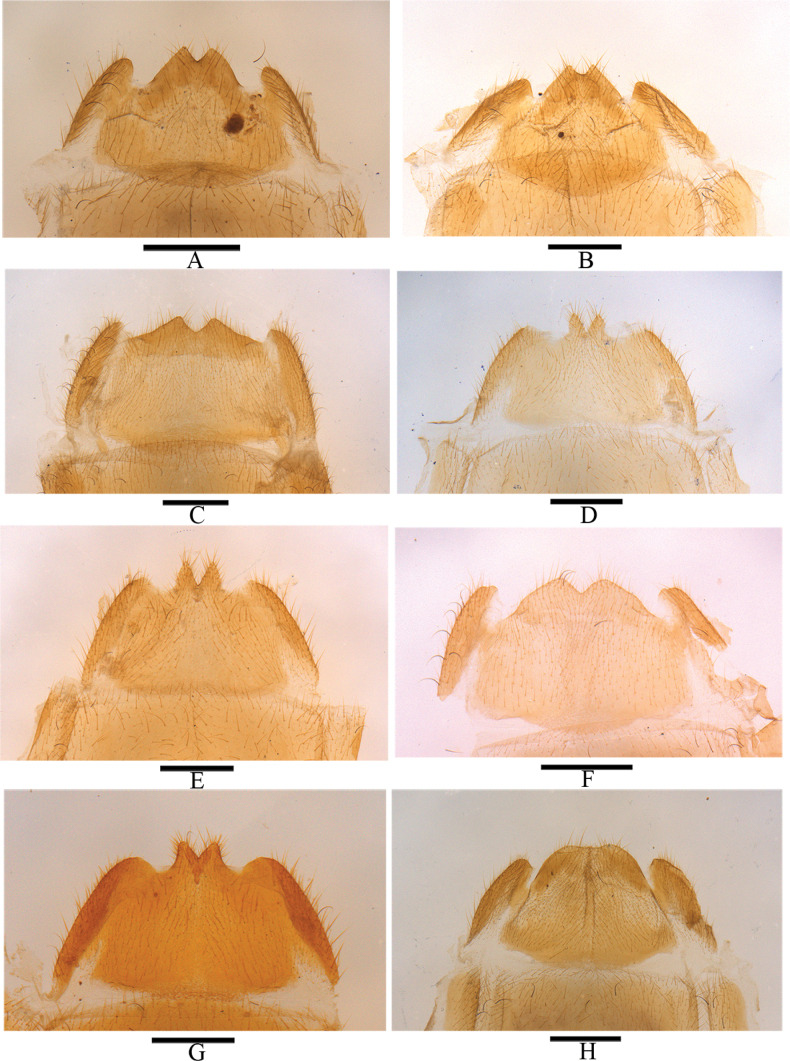
Abdominal sterite VIII of female, ventral view **A***Lycocerushubeiensis* (Švihla, 2004) **B***L.centrochinensis* (Švihla, 2004) **C***L.guerryi* (Pic, 1906) **D***L.genaemaculatus* (Wittmer, 1951) **E***L.flavipennis* sp. nov. **F***L.zdeneki* (Švihla, 2004) **G***L.kubani* (Švihla, 2004) **H***L.laterophysus* sp. nov. Scale bars: 0.5 mm.

**Figure 17.**
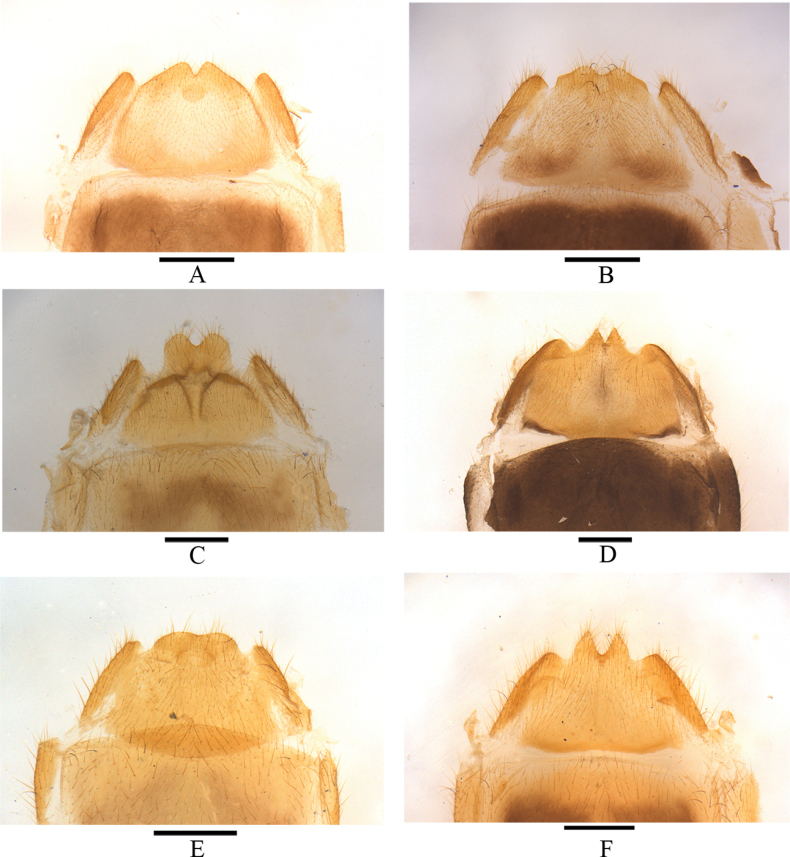
Abdominal sterite VIII of female, ventral view **A***Lycocerusbilineatus* (Wittmer, 1995) **B***L.jelineki* (Švihla, 2004) **C***L.maoershanensis* sp. nov. **D***L.putzi* Švihla, 2011 **E***L.chongqingensis* sp. nov. **F***L.bispermathecus* sp. nov. Scale bars: 0.5 mm.

Abdominal sternite VIII (Fig. 17F): triangular emarginations in middle and on both sides of posterior margin, lateral emarginations feebly deeper than the middle one, the portion between lateral and middle emarginations narrow and acute at apices, obviously extending over apices of latero-apical angles, which are widely triangular.

Body length: 9.0–10.0 mm (9.3 mm in holotype); width: 2.0–2.3 mm (2.0 mm in holotype).

##### Distribution.

China (Ningxia).

##### Etymology.

The specific name is derived from the Latin *spermatike* (sperm-carrying), referring to its distinctive spermatheca, which has two spiral tubes.

## ﻿Discussion

The characters of tarsal claws, which was emphasized by Wittmer (1995) to define the subgenera of former *Athemus* Lewis, 1895 (now a junior synonym of *Lycocerus*), is proven again to be variable even within a species group (Table 1), as noted by Okushima and Hsiao (2017). Since no character was considered valuable enough to define the subgenera, Okushima (2005) proposed to define species groups to subdivide the large genus *Lycocerus* sensu lato.

**Table 1.** The characteristics of tarsal claws of *L.pallidulus* group.

Species	Male fore and mid-legs		Female fore and mid-legs	
	anterior claws	posterior claws	anterior claws	posterior claws
*Lycoceruspallidulus*				
*L.guerryi*				
*L.guerryiatroapicipennis*				
*L.centrochinensis*				
*L.genaemaculatus*				
*L.hubeiensis*				
*L.jelineki*				
*L.bilineatus*				
*L.zdeneki*				
*L.kubani*				
*L.curvatus*				
*L.pictipennis*				
*L.putzi*				
*L.laterophysus* sp. nov.				
*L.flavipennis* sp. nov.				
*L.putzimimus* sp. nov.			Unknown	Unknown
*L.maoershanensis* sp. nov.				
*L.chongqingensis* sp. nov.				
*L.bispermathecus* sp. nov.				

Note: the gray denotes that the claw has a digitiform tooth at base; the white indicates the claw is simple.

**Figure 18.**
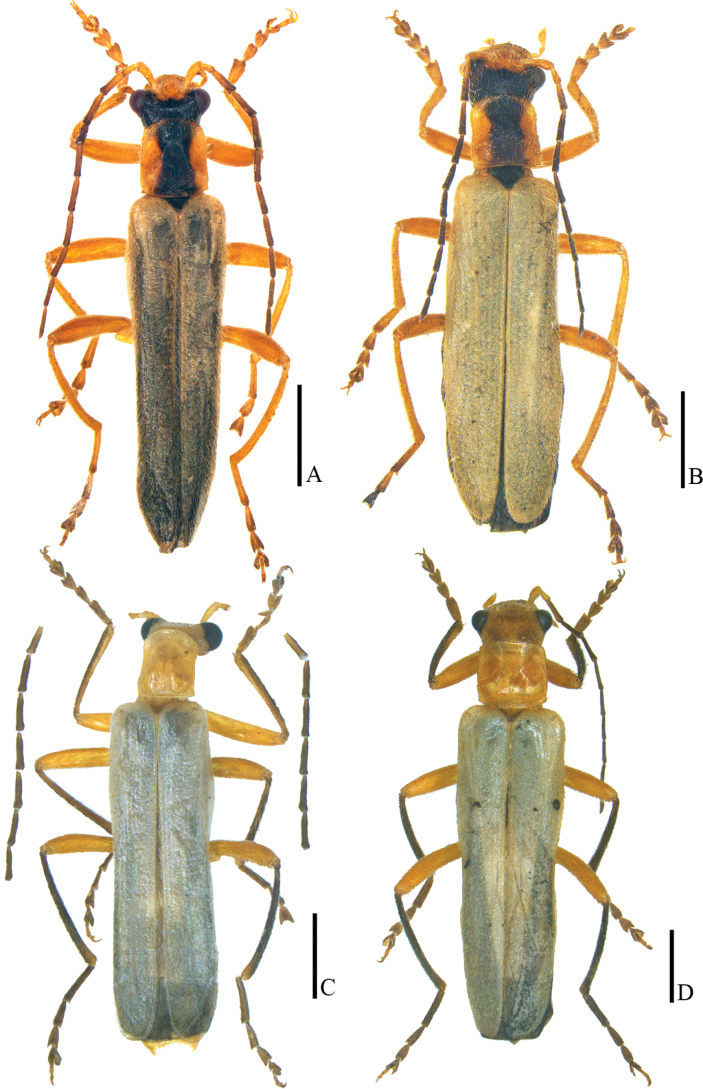
Habitus, dorsal view **A, B***Lycoceruslaterophysus* sp. nov. **C, D***L.flavipennis* sp. nov. **A, C** male **B, D** female. Scale bars: 2.0 mm.

At the beginning, Okushima (2005) defined the species groups of *Lycocerus* based on the genitalia of both sexes. Especially, he highlighted the characters of the female internal organ of reproductive system, including the length of spermathecal duct, and shape, length, and number of tubes of spermatheca. However, in the present study, we discovered that the number of tubes of the spermatheca could be variable within the species group. In the *L.pallidulus* group, the spermatheca of *L.bispermathecus* sp. nov. has two spiral tubes, while all others have only one tube. Also, it is related to *L.centrochinensis* and *L.chongqingensis* sp. nov. on basis of the shape of aedeagus and tarsal claws, as well as the body size and coloration, so they probably belong to a natural species group. In this case, we suggest integrating the characters of both appearance and genitalia to define the species groups of *Lycocerus*, also we should take the distribution range into account.

**Figure 19.**
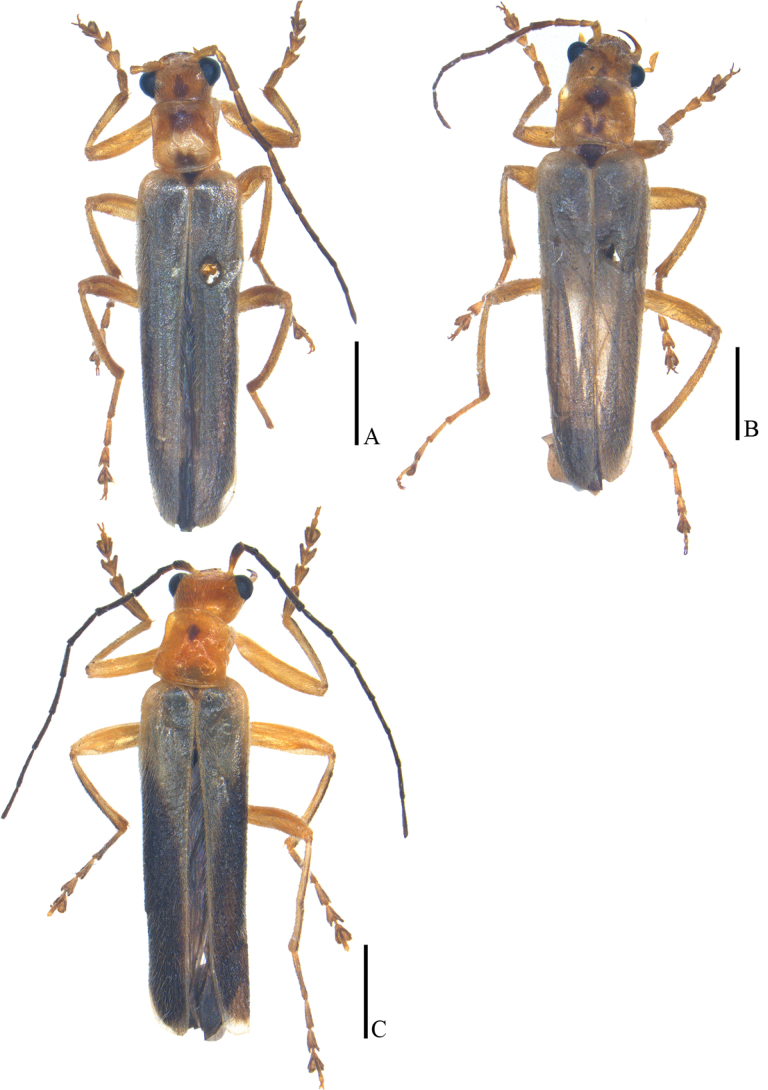
Habitus, dorsal view **A, B***Lycocerusmaoershanensis* sp. nov. **C***L.putzimimus* sp. nov. **A, C** male **B** female. Scale bars: 2.0 mm.

All species of *L.pallidulus* group are distributed in the southern China, located between 21.94–36.60°N and 98.31–21.60°E (Fig. 2). China is located in East Asia and lies in the transitional zone between Palaearctic and Oriental Regions (Zhang 1999). It is a region where some cantharid lineages occur only there and adjacent areas, like other insects (e.g., Bocak and Bocakova 2008). Many lineages with the highest diversity in the Chinese fauna would expand their ranges southwards to the Oriental Region, and *L.pallidulus* group is this case.

**Figure 20.**
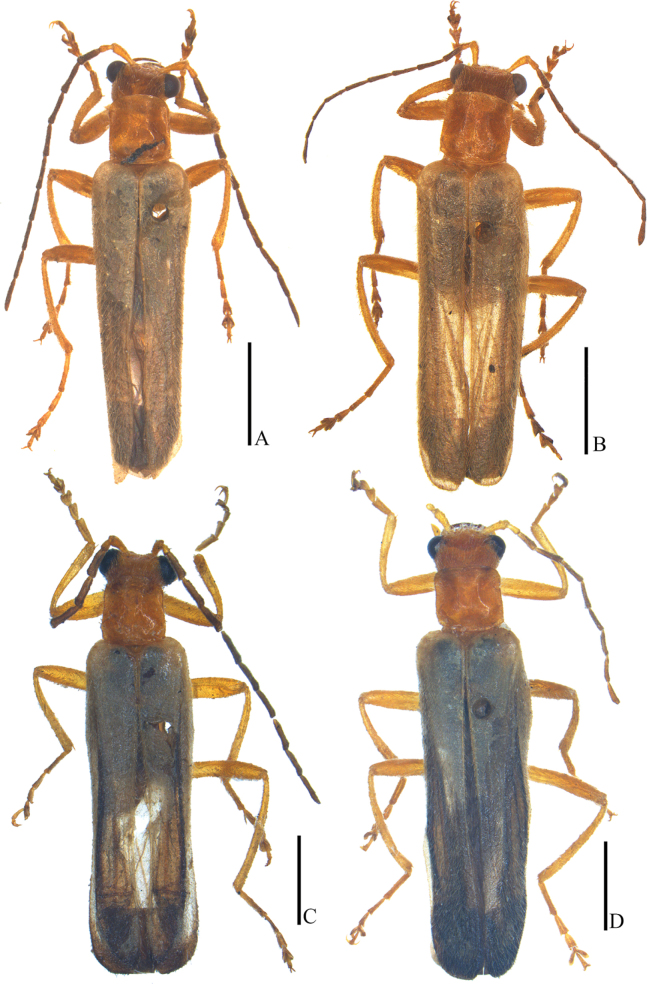
Habitus, dorsal view **A, B***Lycoceruschongqingensis* sp. nov. **C, D***L.bispermathecus* sp. nov. **A, C** male **B, D** female. Scale bars: 2.0 mm.

Originally, *L.pallidulus* group was regarded as a subgroup and placed in *L.maculicollis* group (Okushima 2005). Based on our studies and Wang et al. (2023), we found that *L.pallidulus* group definitely differs from *L.maculicollis* group in the shape of aedeagus, which is unique in the latter group noted previously by Kazantsev (1999). In addition, the former *L.maculicollis* group has already been suggested as a non-monophyletic (Hsiao 2021; Xi et al. 2022; Wang et al. 2022). Thus, we suggest *L.pallidulus* group be treated as an independent species group.

Within the *L.pallidulus* group, the species can be distinguished by their claws (Table 1) and the following key is designed around this feature combined with other morphological characters.

### ﻿Key to the species of *L.pallidulus* group

**Table:** 

1	Elytra bicolored, mixed black with light yellow	**2**
–	Elytra uniformly light yellow	**9**
2	Elytra with black median longitudinal bands	**3**
–	Elytra with black apices	**4**
3	Pronotum uniformly orange; aedeagus: ventral process of each paramere stout and nearly truncated apically in lateral view (Fig. 6C)	***L.bilineatus* (Wittmer, 1995)**
–	Pronotum yellow, dark brown in middle; aedeagus: ventral process of each paramere slender and nearly rounded apically in lateral view (Fig. 11C)	***L.curvatus* (Wittmer, 1995)**
4	Elytra black at apical 2/3, with a long triangular area pale yellow along suture	**5**
–	Elytra black at most at apical 1/5	**6**
5	Pronotum with a small dark brown rounded marking; aedeagus: ventral process of each paramere moderately shorter than dorsal plate, laterophyse furcate at apex (Fig. 9F)	***L.putzimimus* sp. nov.**
–	Pronotum uniformly yellow; aedeagus: ventral process of each paramere much shorter than dorsal plate, laterophyse acute at apex (Fig. 9C)	***L.putzi* Švihla, 2011**
6	All claws simple in male; spermatheca with 2 spiral tubes (Fig. 15B)	***L.bispermathecus* sp. nov.**
–	Fore and mid- anterior and or posterior claws legs each with a digitiform tooth at base in male; spermatheca with 1 spiral tube	**7**
7	Fore and mid- anterior and posterior claws legs each with a digitiform tooth at base in male; aedeagus: ventral process of each paramere nearly straight in ventral view (Figs 4D, 5A), dorsal plate slightly longer or nearly as long as ventral process (Figs 4F, 5C)	**8**
–	Fore and mid-anterior claws each with a digitiform tooth at base in male; aedeagus: ventral process of each paramere distinctly bent inwards in ventral view (Fig. 3A), dorsal plate distinctly longer than ventral process (Fig. 3C)	***L.guerryiatroapicipennis* (Pic, 1914)**
8	Aedeagus: ventral process of each paramere obviously longer than laterophyse (Fig. 4F), the distance between ventral processes wider than that between lateral margins of dorsal plates (Fig. 4E)	***L.hubeiensis* (Švihla, 2004)**
–	Aedeagus: ventral process of each paramere nearly as long as laterophyse (Fig. 5C); the distance between ventral processes narrower than that between lateral margins of dorsal plates (Fig. 5B)	***L.kubani* (Švihla, 2004)**
9	Head bicolored, mixed yellow or orange with black	**10**
–	Head uniformly yellow or orange	**12**
10	Vertex yellow, each side with a black marking around eye; aedeagus: the distance between ventral processes distinctly wider than that between lateral margins of dorsal plates (Fig. 4B)	***L.genaemaculatus* (Wittmer, 1951)**
–	Vertex unlike above, never with markings around eyes; aedeagus: the distance between ventral processes narrower than that between lateral margins of dorsal plates (Figs 8B, 9B)	**11**
11	Head and pronotum yellow, vertex with a triangular dark brown marking in middle, pronotum with two irregular black markings near middle of anterior and posterior margins (Fig. 19A); aedeagus: ventral process of each paramere gradually thinned apically, as long as dorsal plate (Fig. 9C), inner apical angle of dorsal plate acute (Fig. 9B), laterophyse narrow and directing inwards in ventral view (Fig. 9A)	***L.maoershanensis* sp. nov.**
–	Vertex black, clypeus yellow, pronotum yellow, with a black wide median longitudinal band (Fig. 18A); aedeagus: ventral process of each paramere shorter than dorsal plate and expanded near base in lateral view (Fig. 8C), inner apical angle of dorsal plate rounded (Fig. 8B), laterophyse broad and directing outwards in ventral view (Fig. 8A)	***L.laterophysus* sp. nov.**
12	All claws simple in male	**13**
–	Fore and mid-anterior and posterior claws each with a digitiform tooth at base in male	**14**
13	Body larger, 8.0–10.0 mm in length; aedeagus: ventral process of each paramere nearly vertical in lateral view (Fig. 3F), dorsal plates of parameres with inner margins abruptly diverging near middle (Fig. 3E); abdominal sternite VIII strongly narrowed posteriorly, the portion between lateral and middle emarginations triangular at apices, which distinctly extending over apices of latero-apical angles (Fig. 16B)	***L.centrochinensis* (Švihla, 2004)**
–	Body smaller, 7.8–9.0 mm in length; aedeagus: ventral process of each paramere slightly bent ventrally in lateral view (Fig. 10C), dorsal plates of parameres with inner margins feebly protuberant near base (Fig. 10B); abdominal sternite VIII moderately narrowed posteriorly, the portion between lateral and middle emarginations rounded at apices, which slightly extending over apices of latero-apical angles (Fig. 17E)	***L.chongqingensis* sp. nov.**
14	Fore and mid-anterior and posterior claws with a digitiform tooth at base in male	**15**
–	Fore and mid-anterior claws with a digitiform tooth at base in male	**16**
15	Aedeagus: basal piece very large, ~ 3× longer than dorsal plate of each paramere in lateral view (Fig. 8F), dorsal plate narrow (Fig. 8E), laterophyse slightly bent inwards in ventral view (Fig. 8D)	***L.flavipennis* sp. nov.**
–	Aedeagus: basal piece nearly as long as dorsal plate of each paramere in lateral view (Fig. 7F), dorsal plate wide (Fig. 7E), laterophyse obviously bent outwards in ventral view (Fig. 7D)	***L.pictipennis* (Wittmer, 1995)**
16	Fore and mid-anterior and posterior claws each with a digitiform tooth at base in female	**17**
–	Fore and mid-anterior claws each with a digitiform tooth at base in female	***L.jelineki* (Švihla, 2004)**
17	aedeagus: ventral process of each paramere obviously shorter than laterophyse in ventral view (Fig. 4D), laterophyse slender, nearly approaching to each other in ventral view (Fig. 4D), inner apical angle of dorsal plate emarginate at apex (Fig. 4E)	***L.zdeneki* (Švihla, 2004)**
–	aedeagus: ventral process of each paramere obviously longer than laterophyse in ventral view (Fig. 3A), laterophyse stout, nearly directing outward in ventral view (Fig. 3A), inner apical angle of dorsal plate subrounded at apex (Fig. 3B)	**18**
18	Female abdominal sternite VIII with the portions between middle and lateral emarginations wide and rounded at apices (Okushima, 2005: fig. 22d); aedeagus: ventral process of each paramere slightly bent inwards in ventral view (Okushima, 2005: fig. 22a), dorsal plate of each paramere narrow and obviously separate apically (Okushima, 2005: fig. 22c)	***L.pallidulus* (Wittmer, 1995)**
–	Female abdominal sternite VIII with the portions between middle and lateral emarginations narrower and right-angled at apices (Fig. 16C); aedeagus: ventral process of each paramere distinctly bent inwards in ventral view (Fig. 3A), dorsal plate of each paramere slight wide and feebly separate apically (Fig. 3C)	***L.guerryi* (Pic, 1906)**

## ﻿Conclusions

The *Lycoceruspallidulus* subgroup originally placed in *L.maculicollis* group is suggested as an independent species-group herein. This group is mainly distributed in the southern China and easily recognized by the middle-sized body and pale yellow or even transparent elytra, sometimes with black longitudinal bands or markings, as well as the genitalia of both sexes. In total 19 species are currently attributed to this group, including, *L.laterophysus* sp. nov., *L.flavipennis* sp. nov., *L.putzimimus* sp. nov., *L.maoershanensis* sp. nov., *L.chongqingensis* sp. nov. and *L.bispermathecus* sp. nov., discovered from China. These species can be distinguished from one another by the body coloration, structures of tarsal claws in both sexes, shapes of aedeagus and abdominal sternite VIII of female. The results of this study provide a better understanding about the morphological and specific diversities of *Lycocerus*, to improve the classification of this speciose genus.

## Supplementary Material

XML Treatment for Lycocerus pallidulus

XML Treatment for Lycocerus guerryi

XML Treatment for Lycocerus guerryi atroapicipennis

XML Treatment for Lycocerus centrochinensis

XML Treatment for Lycocerus genaemaculatus

XML Treatment for Lycocerus hubeiensis

XML Treatment for Lycocerus kubani

XML Treatment for Lycocerus zdeneki

XML Treatment for Lycocerus bilineatus

XML Treatment for Lycocerus jelineki

XML Treatment for Lycocerus putzi

XML Treatment for Lycocerus pictipennis

XML Treatment for Lycocerus curvatus

XML Treatment for Lycocerus laterophysus

XML Treatment for Lycocerus flavipennis

XML Treatment for Lycocerus maoershanensis

XML Treatment for Lycocerus putzimimus

XML Treatment for Lycocerus chongqingensis

XML Treatment for Lycocerus bispermathecus

## ﻿Additional information

### Author ORCIDs

Haoyu Liu https://orcid.org/0000-0003-1383-5560

Xingke Yang https://orcid.org/0000-0003-3676-6828

Yuxia Yang https://orcid.org/0000-0002-3118-6659

### Data availability

All of the data that support the findings of this study are available in the main text.
